# ERO1A as a novel biomarker for risk stratification and immunotherapeutic guidance in early-stage lung adenocarcinoma

**DOI:** 10.7717/peerj.21347

**Published:** 2026-06-17

**Authors:** Wangyu Chen, Haiming Li, Jie Zeng, Guijing Xie, Zhibo Chen, Xie Xu, Wenjie Bai, Gang Xiao, Jianjiang Xie

**Affiliations:** 1 Department of Thoracic Surgery, Guangzhou First People’s Hospital, South China University of Technology, Guangzhou, China; 2 Department of Thoracic Surgery, Guangzhou First People’s Hospital, Guangdong Medical University, Guangzhou, China; 3 Center for Medical Research on Innovation and Translation, Guangzhou, China

**Keywords:** Early-stage lung adenocarcinoma, ERO1A, Prognostic biomarker, Tumor immune infiltration, Immunotherapy

## Abstract

**Background:**

A considerable proportion of patients with early-stage lung adenocarcinoma (esLUAD) experience recurrence after curative resection, highlighting the need for improved biomarkers. Current biomarkers such as PD-L1 and tumor mutational burden (TMB) have limited predictive utility in early-stage disease. Through an unbiased screening approach, endoplasmic reticulum oxidoreductase 1α (ERO1A) was identified as a candidate gene for further investigation.

**Methods:**

Multi-omics data from The Cancer Genome Atlas (TCGA) and Gene Expression Omnibus (GEO) databases were integrated. Machine learning algorithms (least absolute shrinkage and selection operator (LASSO), random forest (RF), support vector machine (SVM)) were employed to identify core prognostic genes. Functional enrichment, immune microenvironment analysis, and drug sensitivity prediction were conducted. Key findings were validated using immunohistochemistry (IHC) in an independent institutional cohort.

**Results:**

ERO1A was identified as a robust, independent predictor of poor overall and recurrence-free survival in esLUAD. It was significantly upregulated in tumors and associated with enhanced proliferative pathways. Paradoxically, high ERO1A expression correlated with an immune-activated tumor microenvironment, characterized by enriched CD8 + T cell infiltration, reduced M2 macrophages, and increased mature tertiary lymphoid structures. ERO1A-high tumors exhibited elevated TMB and PD-L1 levels, and were associated with superior response to immune checkpoint inhibitors in validation cohorts. Bioinformatic analysis suggested that ERO1A-high tumors might exhibit resistance to conventional chemotherapy while showing potential sensitivity to epidermal growth factor receptor (EGFR)-targeted therapies, though this was not confirmed in clinical validation.

**Conclusions:**

ERO1A expression stratifies lung adenocarcinoma (LUAD) into distinct subsets with divergent therapeutic implications. It represents a promising biomarker for prognostic stratification and immunotherapy selection, supporting its potential clinical utility in guiding peri-operative therapy decisions.

## Introduction

Lung cancer remains the leading global malignancy, with 2.48 million new cases in 2022 (12.4% of all cancers) and 1.06 million in China alone (Bray et al., 2024; Han et al., 2024). Non-small-cell lung cancer (NSCLC) accounts for 85% of cases, and lung adenocarcinoma (LUAD) represents >40%, with incidence continuing to rise (Duma, Santana-Davila & Molina, 2019). Although patients with stage I–II LUAD have a better prognosis than those with advanced disease, 30–55% relapse after curative resection, yielding 5-year survival <60%, underscoring the need for molecular refinement of current staging (Borczuk et al., 2005; Hung et al., 2012; Uramoto & Tanaka, 2014; Potter et al., 2023).

Recent data show that immunotherapy has improved 3-year survival in advanced NSCLC from 26% to 43% (Siegel et al., 2025). Immune-checkpoint inhibitors (ICIs) are now standard first-line therapy for patients with advanced and locally advanced unresectable non-small cell lung cancer (NSCLC) (Herbst, Morgensztern & Boshoff, 2018; Calvo et al., 2021). Yet for patients with resectable early-stage NSCLC, reducing post-operative recurrence remains an unmet need. Three phase III trials—CheckMate-816 (neoadjuvant nivolumab plus chemotherapy), IMpower010 (adjuvant atezolizumab), and AEGEAN (peri-operative durvalumab)—have moved ICIs into the peri-operative setting with breakthrough results: each demonstrated significant increases in pathologic complete response (pCR) and prolonged event-free survival (EFS), leading to Food and Drug Administration (FDA) and National Medical Products Administration (NMPA) approvals for resectable NSCLC (Felip et al., 2021; Forde et al., 2022; Heymach et al., 2023; Mitsudomi et al., 2025; Forde et al., 2025; Felip et al., 2025). Neoadjuvant ICIs in resectable LUAD have shown promising activity (MPR 20–40%), suggesting the potential to induce major pathologic responses, enhance resectability, and curb distant metastasis (Shukla & Hanna, 2021). However, PD-L1 expression and tumor mutational burden (TMB) exhibit limited sensitivity and specificity in early LUAD (Brahmer et al., 2015; Sacher & Gandhi, 2016; Carbone et al., 2017; Hellmann et al., 2018; Garassino et al., 2019; Paz-Ares et al., 2019; McGrail et al., 2021; Li et al., 2021; Yu et al., 2022). This underscores the urgent need for novel biomarkers that can accurately predict both malignant progression risk and response to immunotherapy.

Endoplasmic-reticulum oxidoreductase 1α (ERO1A) is an ER-resident thiol oxidoreductase that catalyzes disulfide-bond formation (Inaba et al., 2010; Zito, Guarrera & Janssen-Heininger, 2024). Pan-cancer data indicate that elevated ERO1A drives proliferation, metastasis, angiogenesis, immune evasion and chemoresistance (Chen et al., 2024c); in LUAD, Xie et al. (2022) demonstrated that ERO1A promotes tumor proliferation and metastasis *via* the Wnt2/β-catenin signaling pathway, while Zhang et al. (2024) similarly showed that knockdown of ERO1A suppresses tumor growth, migration, and invasion by inhibiting the Wnt/β-catenin pathway. ERO1A also sculpts the immune microenvironment: high expression correlates with fewer CD8^+^ T cells and more M2-polarized macrophages (Liu et al., 2021), and its ablation restores CD8^+^ T-cell infiltration while reinvigorating anti-PD-1 responses (Liu et al., 2023). Yet its specific role in early-stage LUAD remains unexplored.

Here, we integrated public databases (TCGA/GEO) and ICI cohorts to systematically evaluate the prognostic and predictive value of ERO1A in early LUAD, with validation in an independent FFPE series. These findings may refine risk stratification and guide immunotherapy decisions for early-stage LUAD.

## Methods

### Public databases and data acquisition

The Tumor Immune Estimation Resource (TIMER2.0) (http://timer.cistrome.org/) (Li et al., 2020) was used to investigate differential expression of ERO1A between tumor and adjacent normal tissues across all TCGA tumors. Gene Expression Profiling Interactive Analysis (GEPIA2) (https://gepia2.cancer-pku.cn/) (Tang et al., 2017) database was utilized to analyze the ERO1A mRNA expression level and prognostic value in LUAD. Protein expression levels were analyzed using the Human Protein Atlas (HPA) database (http://www.proteinatlas.org) (Uhlén et al., 2015).

TCGA-LUAD data was downloaded from the Sangerbox website (http://www.sangerbox.com) (Chen et al., 2024a). These data included information on: RNA-seq (HTSeq-FPKM) and clinical profiles. The ENSG_IDs were converted to gene symbols, and rows with more than 50% zero values as well as columns with more than 50% missing values were removed. The data was then normalized using a log2(X+1) transformation. The clinical cohort was filtered to include only pathological stage I-II LUAD-not otherwise specified (NOS) cases, excluding patients with follow-up <1 month or incomplete survival data, and 239 valid clinical data cases were retained. After integration with expression profile data, a total of 265 samples were included, comprising 233 tumor tissues and 32 paired normal tissues.

The GSE72094, GSE135222 and GSE260770 datasets were downloaded from the GEO database (https://www.ncbi.nlm.nih.gov/geo/). We converted probe IDs to gene names and constructed an expression matrix and grouping information. Among them, the GSE72094 cohort was used to analyze differential expression of ERO1A and its prognostic significance in esLUAD. To validate the role of ERO1A in immunotherapy response, we analyzed two independent cohorts: GSE135222 (*n* = 27, advanced NSCLC, anti-PD-1/PD-L1) and GSE260770 (*n* = 50, early-stage NSCLC, sintilimab), employing differential expression, survival, and receiver operating characteristic (ROC) analyses.

### Differential expression analysis

Patients were assigned to ERO1A-high and ERO1A-low groups based on the median expression value. Differentially expressed genes (DEGs) between the two groups were identified using the Sangerbox website. Genes with *P*-value < 0.05 and |log_2_(FC)| ≥ 1.5 were considered statistically significant and included in further analysis. The resulting DEGs were subjected to Gene Ontology (GO) enrichment analysis to elucidate the ERO1A-related biological processes, molecular functions, and cellular components in esLUAD.

### Identification of prognostic genes in esLUAD

This study adopted a four-level integrated strategy to screen key genes in esLUAD: First, using the “limma” tool on the Sangerbox platform, DEGs between tumor and normal lung tissues were identified with thresholds set at |log_2_(FC)| ≥ 1.5 and *P*-value < 0.05; DEGs were screened accordingly. All DEGs were presented as a volcano plot. Next, bulk survival analysis for DEGs was conducted for both overall survival (OS) and relapse-free survival (RFS), and the intersection of significantly associated genes was taken to identify candidate genes related to survival and recurrence. After annotation *via* the Ensembl database (GRCh38) to remove long non-coding RNAs, three machine learning algorithms were employed for cross-validation, reducing redundant genes through an intersection of results from these algorithms.

Least absolute shrinkage and selection operator (LASSO) regression was implemented using the glmnet R package (version 4.1-8). Ten-fold cross-validation was performed with the cv.glmnet function, and the optimal penalty parameter λ was chosen as lambda.min—the value that minimized the cross-validated deviance. Random forest analysis was conducted with the randomForest R package (version 4.7-1.1). An initial model was built with 500 trees (ntree = 500) and the default number of variables tried at each split (mtry = √p for classification). The optimal number of trees was determined by locating the minimum out-of-bag (OOB) error rate using which.min(rf$err.rate[,1]) (where err.rate[,1] corresponds to the OOB error rate), and a final model was refit with this tree count. Variable importance was assessed by the mean decrease in accuracy (permutation importance). Support vector machine with recursive feature elimination (SVM-RFE) was performed using a custom implementation based on the e1071 R package (version 1.7-14). A linear kernel was employed, and the cost parameter was kept at the default value (C = 1). Five-fold cross-validation was applied across the entire set of candidate genes; in each fold, features were recursively eliminated based on the absolute weights of the linear SVM (with larger absolute weights indicating greater contribution to classification), and the model was retrained. The final feature set was selected as the number of top-ranked genes that minimized the average cross-validation error across the five folds.

The intersection of genes identified by all three algorithms—ERO1A, PLEK2, CA4, and RS1—was considered the most robust set of candidate biomarkers. Finally, univariate and multivariate Cox regression analyses were used to identify potential core prognostic markers in esLUAD.

### Functional enrichment analysis

To comprehensively analyze the molecular mechanisms of ERO1A in esLUAD, we used a combination of GO enrichment analysis and Gene Set Enrichment Analysis (GSEA). The R package ClusterProfiler was used to assess the GO functions potentially enriched by DEGs between ERO1A-high and ERO1A-low groups, in order to better understand the cancer-related processes involving ERO1A-associated genes. GSEA of various gene signatures was performed using the R ClusterProfiler package. These gene sets were obtained based on correlations (|Pearson R| ≥ 0.3 and *P*-value < 0.05); compared with the results produced by GO analysis, the outcomes from GSEA are considered more reliable because GSEA takes into account the entire genome-wide expression profile and does not require setting a threshold for identifying differentially expressed genes.

### Immune infiltration analysis

To comprehensively characterize the tumor immune microenvironment (TIME) in relation to ERO1A expression, we performed immune cell infiltration analysis using a multi-algorithm deconvolution approach on transcriptomic data from the TCGA-esLUAD cohort. Early-stage samples were analyzed separately to account for potential stage-specific immune contexture. The R package immunedeconv (v2.1.0) was employed to integrate eight algorithms to estimate immune cell abundances: CIBERSORT, xCell, quanTIseq, EPIC, ConsensusTME, ABIS, TIMER, and ESTIMATE. Each algorithm was run using default parameters and reference signatures specific to human immune cell types. Spearman’s rank correlation was computed between ERO1A expression and each estimated immune cell fraction across all samples. Only correlations with a false discovery rate (FDR)-adjusted *P*-value < 0.05 were considered statistically significant. Results were visualized using scatter plots.

For key immune subgroups, using the TCGA-esLUAD database, a correlation analysis was performed between the expression of ERO1A and immune cell markers. A positive correlation was defined as Spearman’s ρ > 0 with *P* < 0.05; a negative correlation was defined as ρ < 0 with *P* < 0.05.

### Immunotherapy response prediction

Somatic mutation data (MAF files) were downloaded from The Cancer Genome Atlas (TCGA) database and processed using the maftools R package. Spearman’s correlation was used to evaluate the association between ERO1A expression and TMB. We analyzed the correlation between ERO1A expression and a curated set of immune checkpoint genes in esLUAD using Pearson’s correlation. Correlation matrices were visualized with the R package corrplot.

### Drug sensitivity analysis

To investigate the impact of ERO1A expression levels on drug sensitivity in esLUAD, drug sensitivity was predicted using the oncoPredict R package (v0.2). Genomics of Drug Sensitivity in Cancer (GDSC2) database (https://www.cancerrxgene.org/) was used as the reference dataset for model training, which contains drug sensitivity data (IC50 values) and corresponding gene expression profiles from a large panel of cancer cell lines spanning multiple tumor types. Of note, GDSC2 is derived from cancer cell lines rather than patient tumor specimens, and therefore does not include drug response data from esLUAD patient samples. Gene expression data from the TCGA-esLUAD cohort were then used as the target dataset to infer drug sensitivity based on the trained model. Samples were stratified into ERO1A-high and ERO1A-low groups based on median expression. Differential drug sensitivity between groups was assessed using the Wilcoxon rank-sum test on log2-transformed half-maximal inhibitory concentration (IC_50_) values. The lower the IC50 value, the higher the sensitivity, indicating that the drug has a significant inhibitory effect at a lower concentration.

### Clinical specimens

We obtained tissue specimens from 153 patients who underwent pulmonary resection surgery at Guangzhou First People’s Hospital between 2022 and 2023. Among these, patients diagnosed with LUAD were assessed for eligibility. The inclusion criteria were as follows: (a) surgical resection; (b) pathologically confirmed TNM stage I–II or pre-stage LUAD; (c) complete clinicopathological information, follow-up data, and chest CT data. Recurrence was defined as lesions confirmed by clinical and radiological findings, occurring at least 1 year after surgery. After applying criteria, a total of 99 patients with esLUAD were included. All data were collected at diagnosis or during subsequent follow-up. Ethical approval and patient consent: This study was approved by the institutional ethics committee of Guangzhou First People’s Hospital (No. S-2024-255-02). The requirement for informed consent was waived due to the retrospective nature of the study. Clinical characteristics are summarized in Table S1. Detailed protocols for immunohistochemistry (IHC) staining and scoring, as well as the summarized data for immune cell subsets (Table S2), are provided in Note S1.

### Statistical analysis

Results are presented as mean ± standard deviation. Categorical variables were analyzed with chi-square or Fisher’s exact test. Correlation analyses used Spearman or Pearson methods as appropriate. ROC curve analysis was used to assess the diagnostic performance of ERO1A in LUAD patients. Prognostic analyses in this study included Kaplan-Meier (KM) analysis with hazard ratio (HR) calculated using the log-rank test, along with time-dependent ROC curve evaluation. In addition, univariate and multivariate Cox regression analyses were conducted to identify independent prognostic factors for esLUAD.

Data processing and statistical analysis were conducted using GraphPad Prism 10 and R (version 4.2.2). All statistical tests were two-sided. *P* values for each analysis are indicated in the figures, with ns, not significant, *P < 0.05, **P < 0.01, or ***P < 0.001 considered statistically significant.

## Results

### Study workflow

**Figure 1:**
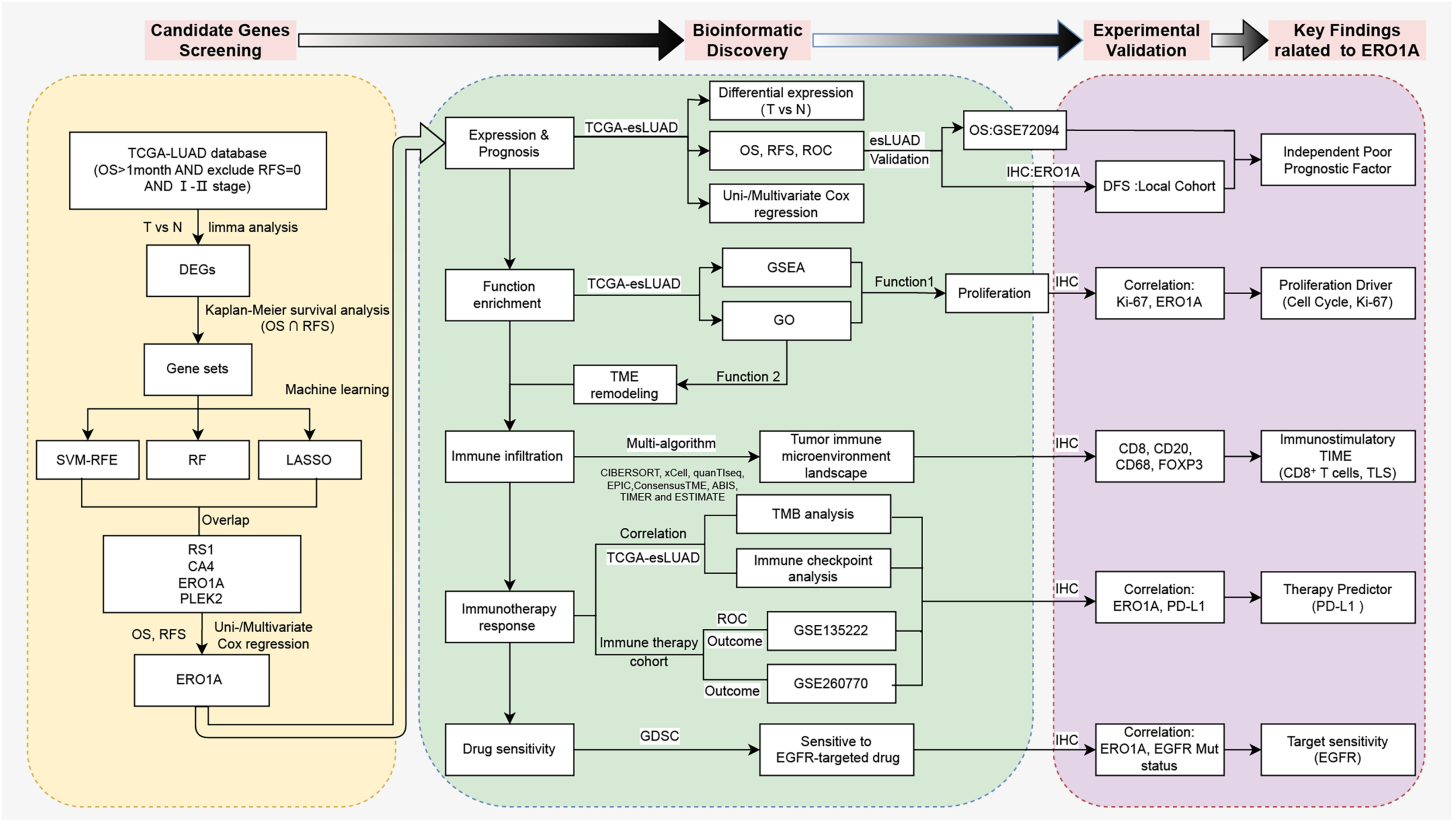
Study workflow.

The study workflow is summarized in Fig. 1.

### Machine-learning integration identifies ERO1A as an independent predictor of overall and recurrence-free survival in early-stage LUAD

**Figure 2:**
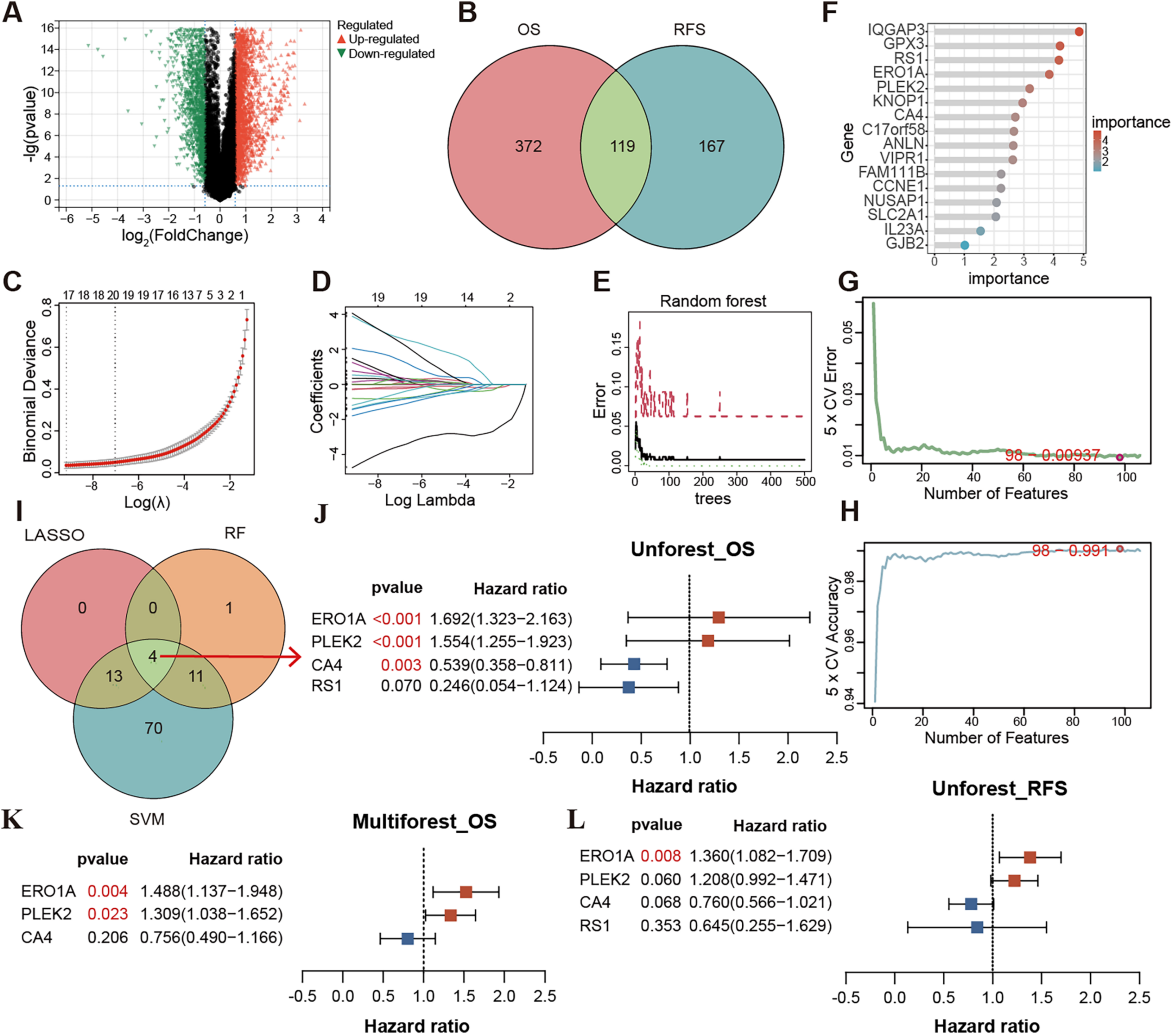
Identification of core genes in esLUAD. (A) Volcano plot displaying DEGs between tumor (T) and adjacent normal (N) tissues in the TCGA-esLUAD cohort. DEGs were identified using the limma package with thresholds of |log_2_(FC)| > 1.5 and *P*-value < 0.05. (B) Venn diagram showing the overlap between genes significantly associated with overall survival (OS) and recurrence-free survival (RFS) among the DEGs. (C–H) Feature selection from prognosis-associated DEGs using three complementary machine learning algorithms to identify robust biomarkers. LASSO regression (C, D) applies L1 regularization to shrink regression coefficients, reducing overfitting and selecting features with the strongest prognostic associations—(C) shows cross-validation error to determine optimal penalty parameter (λ), (D) displays coefficient shrinkage as λ increases. Random Forest (E, F) is an ensemble method that evaluates variable importance through permutation accuracy reduction—(E) shows error stabilization with increasing trees, (F) ranks genes by importance score (mean decrease in accuracy). SVM with recursive feature elimination (G, H) identifies optimal feature subsets through cross-validation—(G) shows cross-validation error across feature numbers, (H) displays corresponding accuracy. (I) The intersection of genes selected by all three algorithms (RS1, CA4, ERO1A, PLEK2) represents the most robust prognostic candidates, as consensus across algorithms reduces algorithm-specific bias and false positives. (J–L) Forest plots of univariate and multivariate Cox regression analyses evaluating the prognostic value of the four hub genes for (J) OS (univariate), (K) OS (multivariate), and (L) RFS (univariate). Abbreviations: DEGs, differentially expressed genes; T, tumor tissues; N, adjacent normal tissues; TCGA, the cancer genome atlas; esLUAD, early-stage lung adenocarcinoma; OS, overall survival; RFS, recurrence-free survival; LASSO, least absolute shrinkage and selection operator; RF, random forest; SVM, support vector machine.

To identify genes associated with the prognosis of early-stage lung adenocarcinoma (esLUAD), we first performed differential expression analysis on TCGA-esLUAD RNA-seq data relative to matched normal lung tissue. Applying thresholds of |log_2_(FC)| ≥ 1.5 and *P* < 0.05 yielded 5,140 DEGs, comprising 2,919 up-regulated and 2,221 down-regulated genes in esLUAD (Fig. 2A). Survival analysis revealed that, among these DEGs, 491 were associated with overall survival (OS), 286 with recurrence-free survival (RFS), and 119 with both endpoints (Fig. 2B). After excluding 13 long non-coding RNAs, 106 protein-coding genes remained for downstream interrogation.

To pinpoint the most robust biomarkers, we performed three complementary machine-learning algorithms with cross-validation: (1) LASSO regression identified 17 key genes (Figs. 2C, 2D); (2) random forest (RF) selected 16 genes (Figs. 2E, 2F); and (3) support vector machine (SVM) retained 98 genes (Figs. 2G, 2H). The intersection of these three approaches yielded four core genes: ERO1A, PLEK2, CA4, and RS1 (Fig. 2I). Univariate Cox regression demonstrated that CA4 conferred a protective effect on OS (*P* < 0.05, HR < 1), whereas ERO1A and PLEK2 were significant risk factors (*P* < 0.05, HR > 1) (Fig. 2J). Multivariate Cox analysis, however, revealed that CA4 did not retain independent prognostic significance for OS (*P* = 0.206), whereas ERO1A and PLEK2 remained significant adverse predictors (Fig. 2K). In the context of RFS, only ERO1A demonstrated independent prognostic value (*P* = 0.008, HR > 1) (Fig. 2L). Collectively, these data indicate that ERO1A is a pivotal determinant of both prognosis and recurrence in esLUAD.

### Expression pattern, early-detection utility, and prognostic value of ERO1A in lung adenocarcinoma

**Figure 3:**
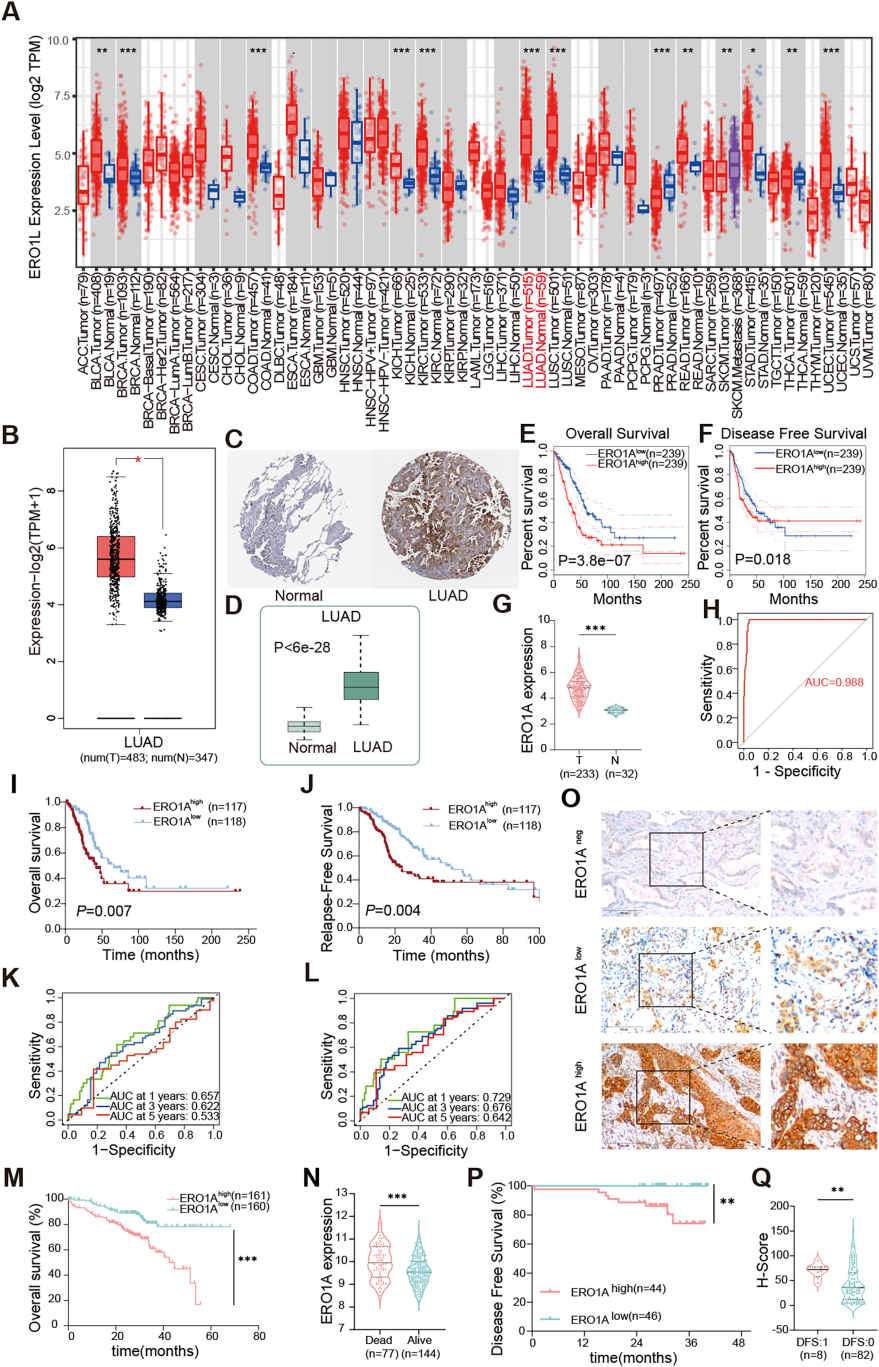
Expression and prognostic value of ERO1A in esLUAD. (A) Pan-cancer analysis of ERO1A expression between tumor and normal (or primary and metastatic) tissues from the TIMER database. (B) ERO1A expression in LUAD and normal tissues based on TCGA and GTEx data (GEPIA). ***P < 0.001; unpaired Student’s t-test. (C) Representative immunohistochemical (IHC) staining images of ERO1A in normal and LUAD tissues from the Human Protein Atlas (HPA) database. (D) ERO1A protein expression in normal lung tissues and LUAD tissues based on the HPA database. (E, F) Survival analysis (GEPIA) of ERO1A expression in TCGA-LUAD: (E) overall survival (OS, *P* < 0.001), (F) disease-free survival (DFS, *P* = 0.018). (G) ERO1A mRNA expression in TCGA-esLUAD tumors (*n* = 233) *vs* normal tissues (*n* = 32). ***P < 0.001; unpaired t-test. (H) Receiver operating characteristic (ROC) curve evaluating the performance of ERO1A expression in discriminating tumor from normal tissue (AUC = 0.988). (I, J) Kaplan-Meier survival analysis in TCGA-esLUAD based on ERO1A expression: (I) OS (*P* = 0.007), (J) relapse-free survival (RFS, *P* = 0.004); log-rank test. (K, L) Time-dependent ROC (tROC) curves predicting (K) OS and (L) RFS at 1, 2, and 3 years in the TCGA-esLUAD cohort. (M) Kaplan-Meier analysis of OS in the GSE72094 esLUAD cohort stratified by ERO1A expression. ***P < 0.001; log-rank test. (N) ERO1A expression between alive (*n* = 144) and deceased (*n* = 77) patients in the GSE72094 esLUAD cohort. ***P < 0.001; unpaired t-test. (O) Representative images of ERO1A IHC staining in our institutional cohort of esLUAD tissue sections (*n* = 90); right panel shows a magnified view of the region indicated by the box. Scale bar: 200 μm. (P) DFS analysis based on ERO1A high/low expression groups defined by IHC H-Score from our cohort. *P* = 0.002, log-rank test. (Q) Comparison of ERO1A H-Score between patients with (*n* = 8) and without (*n* = 82) DFS events. *P* = 0.008; Mann-Whitney U test. Abbreviations: esLUAD, early-stage lung adenocarcinoma; TIMER, tumor immune estimation resource; LUAD, lung adenocarcinoma; TCGA, the cancer genome atlas; GTEx, genotype-tissue expression; GEPIA, gene expression profiling interactive analysis; IHC, immunohistochemistry; HPA, human protein atlas; OS, overall survival; DFS, disease-free survival; ROC, receiver operating characteristic; AUC, area under the curve; RFS, relapse-free survival; tROC, time-dependent receiver operating characteristic; H-score, histochemistry score. ***P < 0.001; **P < 0.01; *P < 0.05; ns, not significant.

To obtain an initial overview, we examined ERO1A across multiple cancer types. In most tumors, ERO1A mRNA was markedly higher than in corresponding normal tissues; notably, in skin cutaneous melanoma (SKCM), metastatic lesions expressed higher levels than primary tumors, suggesting that ERO1A may contribute to various aspects of tumor progression (Fig. 3A). Building on this pan-cancer observation, we focused our subsequent analyses on LUAD. By integrating TCGA-LUAD tumors with TCGA-normal and GTEx-normal lung samples through GEPIA2, we found that ERO1A (also annotated as ERO1L) transcripts were significantly up-regulated in tumors (Fig. 3B). Immunohistochemistry (IHC) data from the Human Protein Atlas further revealed that normal lung parenchyma was almost devoid of ERO1A protein, whereas virtually all LUAD specimens exhibited positive staining with intensities ranging from weak to strong, resulting in a significantly higher overall protein level than normal controls (Fig. 3C, Table S3; with quantitative data shown in Fig. 3D). Survival analyses of the TCGA-LUAD cohort demonstrated that high ERO1A expression was associated with shorter overall survival (Fig. 3E) and disease-free survival (Fig. 3F), underscoring its potential prognostic relevance.

Next, we explored whether ERO1A retained similar properties in esLUAD. In the TCGA-esLUAD dataset, tumor tissues exhibited significantly higher ERO1A mRNA levels than normal lung tissues (Fig. 3G). ROC curve analysis yielded an AUC of 0.988 for distinguishing esLUAD from normal lung tissue, indicating excellent sensitivity and specificity for early detection (Fig. 3H). Survival analyses again revealed that elevated ERO1A correlated with worse OS (Fig. 3I) and recurrence-free survival (RFS, Fig. 3J). When stratified by follow-up interval, the AUCs for 1-, 3-, and 5-year OS were 0.657, 0.662, and 0.533, respectively (Fig. 3K); the corresponding AUCs for RFS were 0.729, 0.676, and 0.642 (Fig. 3L). The progressive decline in AUC suggests that ERO1A is a stronger predictor of early rather than late events.

To validate these results independently, we extracted stage I–II samples from the GEO cohort GSE72094. Within this early-stage subset, high ERO1A expression again predicted poorer OS (Fig. 3M) and was elevated in tumors from deceased patients (Fig. 3N). Finally, IHC analysis of our in-house paraffin-embedded esLUAD specimens demonstrated ubiquitous ERO1A expression throughout tumor regions, with staining intensity ranging from weak to strong (Fig. 3O). Notably, patients exhibiting higher ERO1A expression showed significantly shorter disease-free survival (Fig. 3P). Furthermore, comparative analysis revealed that ERO1A expression levels were markedly elevated in patients who experienced DFS events compared to those who remained recurrence-free (Fig. 3Q).

Taken together, data spanning public databases and independent patient samples consistently demonstrate that ERO1A is significantly up-regulated in esLUAD, possesses high accuracy for early detection, and reliably predicts adverse prognosis.

### ERO1A as an independent prognostic biomarker and indicator of pathologic stage in esLUAD

To systematically evaluate the independent prognostic value of ERO1A, we conducted univariate and multivariate Cox regression analyses in the TCGA-esLUAD cohort, adjusting for age, sex, smoking history, pathologic stage, and other relevant clinical variables, to determine the independent prognostic value of ERO1A. Univariate analyses revealed that age, pathologic stage, and ERO1A expression were significantly associated with OS (Fig. S1A), whereas pathologic stage, T stage, and ERO1A expression were associated with RFS (Fig. S1B). In the fully adjusted multivariate model, pathologic stage (OS: HR = 2.586, 95% CI [1.644–4.067], *P* < 0.001; RFS: HR = 1.726, 95% CI [1.112–2.678], *P* = 0.015) and high ERO1A expression (OS: HR = 1.548, 95% CI [1.209–1.983], *P* < 0.001; RFS: HR = 1.303, 95% CI [1.036–1.638], *P* = 0.024) remained independent risk factors for adverse outcomes in esLUAD (Figs. S1C, S1D).

Collectively, these data establish ERO1A as an independent prognostic factor in esLUAD, supporting its potential utility for risk stratification and therapeutic planning.

### Gene ontology enrichment analysis implicates ERO1A in proliferation and microenvironment dynamics in early-stage LUAD

**Figure 4:**
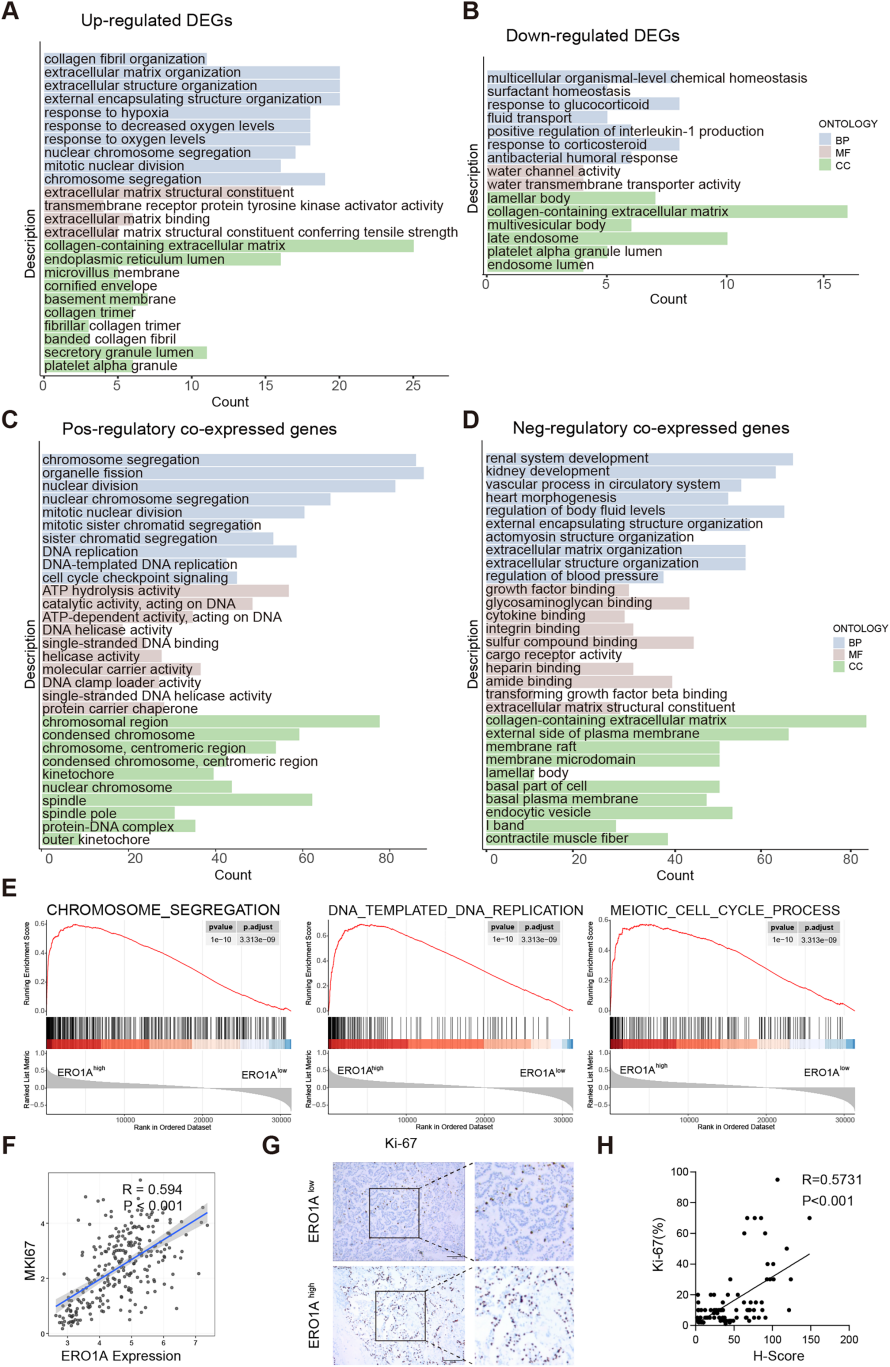
Functional enrichment analysis of ERO1A-associated genes in esLUAD. (A, B) Gene Ontology (GO) analysis of biological processes (BP), cellular components (CC), and molecular functions (MF) for differentially expressed genes (DEGs) identified by comparing ERO1A-high *vs* ERO1A-low groups in the TCGA-esLUAD cohort (|log2(FC)| ≥ 1.5, *P*-value < 0.05). (A) Shows enriched terms for genes up-regulated in the ERO1A-high group (*n* = 185), while (B) shows enriched terms for genes down-regulated in the ERO1A-high group (*n* = 164). (C, D) Functional annotation of ERO1A co-expressed genes in the TCGA-esLUAD cohort. (C) Shows GO terms enriched for genes positively correlated with ERO1A (*n* = 2,297), and (D) shows GO terms enriched for genes negatively correlated with ERO1A (*n* = 1,992). (E) Gene set enrichment analysis (GSEA) of ERO1A co-expressed genes shows significant enrichment in cell proliferation-related pathways. (F) Scatter plot showing the correlation between ERO1A and MKI67 mRNA expression in the TCGA-esLUAD cohort. *P* < 0.001; Spearman’s correlation test. (G) Representative immunohistochemical images of ERO1A and Ki-67 from our institutional esLUAD cohort. The right panel shows a magnified view of the boxed region. Scale bar: 200 μm. (H) Scatter plot showing the correlation between ERO1A protein expression (H-Score) and Ki-67 positive rate in our institutional esLUAD cohort (*n* = 93). Pearson’s correlation coefficient and *P*-value are indicated. Pearson’s correlation test.

To identify ERO1A-associated biological pathways in esLUAD, we conducted Gene Ontology (GO) enrichment analysis of the TCGA-esLUAD cohort. This analysis examined DEGs between tumors with high *vs* low ERO1A expression and genes whose expression correlated with ERO1A levels across the cohort. Analysis of genes significantly upregulated in tumors with high ERO1A expression revealed predominant enrichment in processes critical for cell division, including nuclear chromosome segregation, mitotic nuclear division, and DNA replication (Fig. 4A). Conversely, genes downregulated in these high expressers were enriched for terms related to homeostasis, inflammatory regulation, and specific cellular components. Notably, this included the suppression of positive regulation of interleukin-1 production—a cytokine that paradoxically promotes immunosuppression and tumor progression in the cancer microenvironment (Millares et al., 2018; Wong et al., 2020; Aggen et al., 2021) (Fig. 4B).

Supporting the proliferative association, genes exhibiting a strong positive correlation with ERO1A expression across all esLUAD tumors were robustly enriched in key cell cycle events, notably chromosome segregation, organelle fission, and DNA replication (Fig. 4C). Molecular functions including ATP hydrolysis activity and DNA helicase activity, along with cellular components like kinetochore and condensed chromosome, further emphasized ERO1A’s role in proliferation. This link between ERO1A expression and core proliferative machinery was further validated by Gene Set Enrichment Analysis (GSEA), which confirmed significant associations with chromosome segregation, DNA templated DNA replication, and meiotic cell cycle process in high ERO1A expressers (Fig. 4E).

Genes exhibiting a negative correlation with ERO1A expression levels showed notable enrichment in immune-regulatory pathways, including transforming growth factor beta binding (TGF-β binding), cytokine binding, and growth factor binding (Fig. 4D). TGF-β is a well-established immunosuppressive factor in the tumor microenvironment (TME) (Plaschka et al., 2022). Combined with the observed loss of immune-related processes (antibacterial humoral response) in low ERO1A expressers (Fig. 4B) and enrichment in structural organization pathways (ECM organization, external encapsulating structure organization), these findings strongly suggest ERO1A’s significant role in remodeling the esLUAD TME with potential implications for immune regulation.

To further validate the proliferative role of ERO1A, we assessed its correlation with the well-established proliferation marker *MKI67* in the TCGA-esLUAD cohort. Notably, *ERO1A* expression exhibited a strong positive correlation with *MKI67* (R = 0.594, *P* < 0.001; Fig. 4F). IHC analysis of our institutional esLUAD cohort confirmed that elevated ERO1A protein levels (H-Score) were positively correlated with increased Ki67 positivity (Figs. 4G, 4H).

### ERO1A expression promotes an immune-activated tumor microenvironment and enhances tertiary lymphoid structure maturation in esLUAD

**Figure 5:**
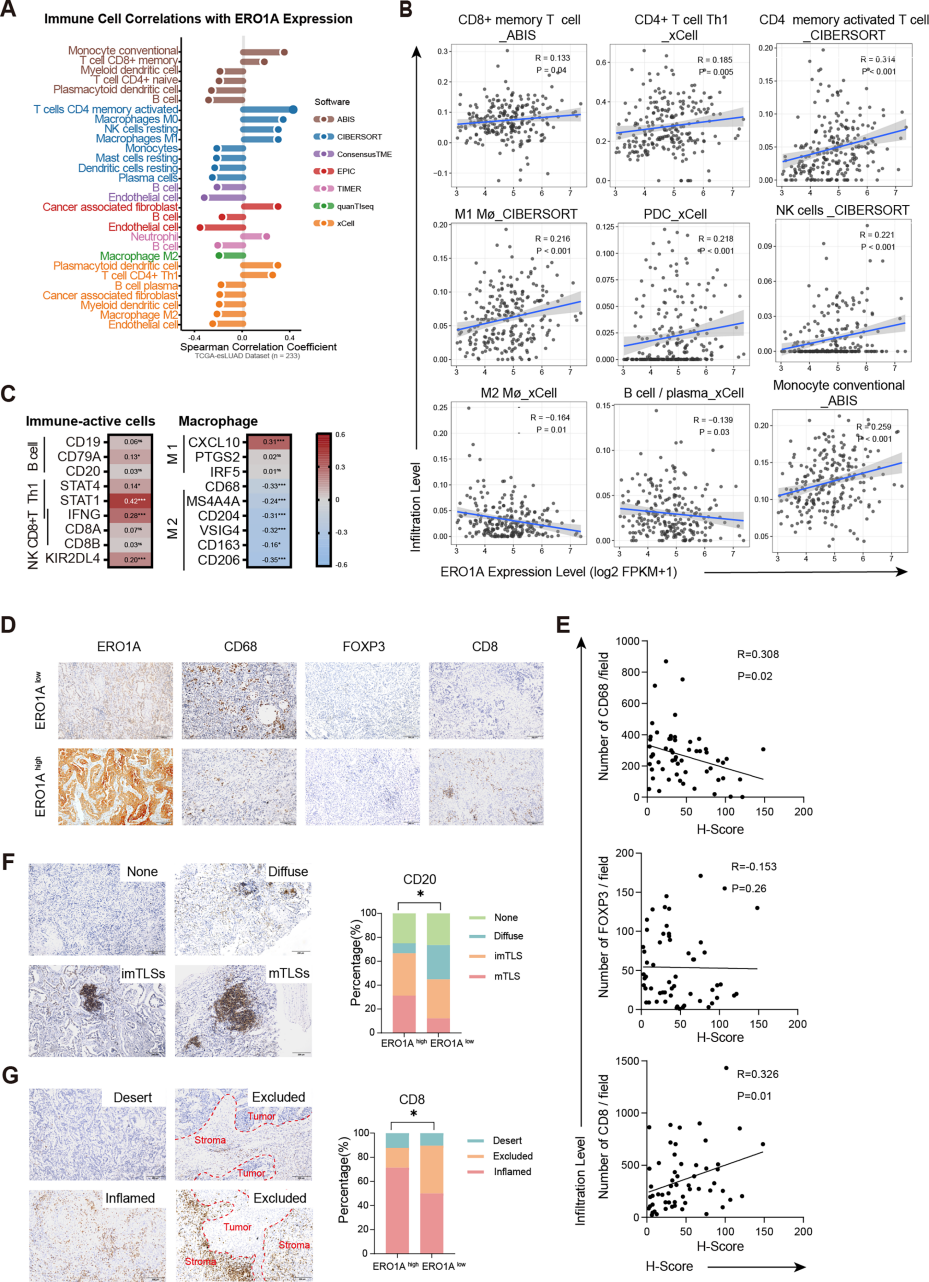
Immune microenvironment characteristics associated with ERO1A in patients with esLUAD. (A) Correlation analysis between ERO1A expression and immune cell infiltration levels in the TCGA esLUAD cohort. The x axis shows Spearman’s correlation coefficient, with positive values indicating a positive association and negative values indicating a negative association. The y axis lists immune cell types. Each bar represents the result from a different deconvolution algorithm, as indicated by the color key (*e.g*., xCell, EPIC, CIBERSORT, quanTIseq, TIMER, ABIS, ConsensusTME, and ESTIMATE). Only correlations with FDR adjusted *P* < 0.05 are displayed, ensuring that only statistically significant associations are presented for interpretation. (B) Scatter plots showing correlations between ERO1A expression and immune infiltration levels estimated by different algorithms in the TCGA-esLUAD cohort. Spearman’s correlation test. (C) Heatmap showing correlations between ERO1A expression and marker genes of specific immune cells in the TCGA-esLUAD cohort. Values represent Spearman correlation coefficients; *P < 0.001, ***P < 0.05, ns, not significant. (D) Representative IHC images of ERO1A, CD68 (macrophages), FOXP3 (regulatory T cells, Tregs), and CD8 (CD8 + T cells) in ERO1A-high and ERO1A-low esLUAD tissues. Scale bar: 200 μm. (E) Scatter plots showing correlation between ERO1A protein expression (H-Score) and infiltration levels of CD68 + macrophages, FOXP3 + Tregs, and CD8 + T cells in our institutional esLUAD cohort (*n* = 57). Spearman’s correlation test. (F) Left: representative IHC images of CD20 used to classify tertiary lymphoid structures (TLS) as none, diffuse, immature TLS (imTLS), or mature TLS (mTLS). Right: proportion of TLS maturation states in ERO1A-high *vs* ERO1A-low esLUAD tissues. *P* = 0.03; chi-square test. Scale bar: 200 μm. (G) Left: representative IHC of CD8 used to classify immune phenotypes as desert (immune desert, lacking CD8 + T cell infiltration in the tumor parenchyma), excluded (immune excluded, CD8 + T cells confined to the tumor stroma but not invading the tumor parenchyma), or inflamed (immune inflamed, with CD8 + T cells infiltrating the tumor parenchyma). Right: distribution of CD8 + T cell infiltration patterns in ERO1A-high *vs* ERO1A-low groups. *P* = 0.04; chi-square test. Scale bar: 200 μm.

To investigate the impact of ERO1A expression on the tumor immune microenvironment, we stratified the TCGA-esLUAD cohort based on ERO1A expression levels and evaluated immune infiltration using multiple deconvolution algorithms, including ABIS, CIBERSORT, TIMER, and xCell, implemented *via* the immunedeconv package. As summarized in Fig. 5A, results from at least one algorithm indicated significant enrichment of CD8^+^ T cells and M1 macrophages in the high ERO1A group, whereas B cells, endothelial cells, and M2 macrophages were more abundant in the low expression group. Infiltration of tumor-associated macrophages and plasmacytoid dendritic cells showed inconsistent correlations with ERO1A expression across different algorithms. These trends were further illustrated for selected cell types in Fig. 5B. Consistent with the above, in esLUAD, high ERO1A expression correlated positively with antitumor immune cells such as CD8^+^ memory T cells, Th1 CD4^+^ T cells, and M1 macrophages, and negatively with immunosuppressive cells including M2 macrophages. A negative correlation was also observed with B cells and plasma cells. Although generally associated with humoral antitumor immunity, both cell types have been implicated in immunosuppressive functions, such as regulatory B cells (Bregs) secreting IL-10, or IgG4-positive plasma cells inhibiting antitumor immune responses (Karagiannis et al., 2013; Sarvaria, Madrigal & Saudemont, 2017).

We next examined the correlations between ERO1A and marker genes of representative immune cells (Fig. 5C). Marker genes for CD8^+^ T cells, Th1 cells, and M2 macrophages were consistent with the predictions from deconvolution analysis. In contrast, B cell markers showed an overall weak positive correlation with ERO1A, contrasting with the deconvolution results. Among M1 macrophage markers, only CXCL10 exhibited a strong positive correlation with ERO1A. It is noteworthy that CXCL10 is not specific to M1 macrophages, as it is also expressed by Th1 and NK cells (Liu, Guo & Stiles, 2011), both of which differed between ERO1A expression groups. By comparison, IRF5, a transcription factor more specifically associated with M1 polarization, showed no significant correlation with ERO1A, suggesting that the observed difference in CXCL10 may reflect contributions from non-M1 cellular sources rather than actual changes in M1 macrophage abundance. Conversely, M2 markers such as CD163 and CD206, along with the pan-macrophage marker CD68, were negatively correlated with ERO1A.

To validate these findings, we performed immunohistochemical staining of key immune markers and correlated their expression with ERO1A (Fig. 5D). High ERO1A expression was associated with reduced infiltration of total macrophages (CD68^+^) and increased CD8^+^ T cell infiltration. No difference was observed in regulatory T cell (Treg, FOXP3^+^) infiltration between ERO1A expression groups (Fig. 5E). Notably, CD20^+^ B cells were rarely found as isolated cells but frequently formed tertiary lymphoid structure (TLS)-like clusters. Based on CD20 distribution patterns and morphological assessment, esLUAD samples were categorized into four groups (Fig. 5F): None (minimal CD20), Diffuse (scattered cells), imTLS (immature TLS, typically appearing as a dense aggregate of CD20^+^ cells without a discernible pale unstained core), and mTLS (mature TLS, characterized by a more organized structure that includes a distinct central area of loosely arranged, CD20-negative cells, morphologically consistent with a putative PDC-rich zone). Tissues with high ERO1A expression showed a significant difference in CD20 distribution, with mature TLS being notably more prevalent. Finally, immune subtyping based on CD8 staining revealed a higher proportion of immune-activated profiles in ERO1A-high samples, and a positive significant difference in subtype distribution was detected between groups (Fig. 5G).

### ERO1A predicts response to immune checkpoint inhibition in esLUAD

**Figure 6:**
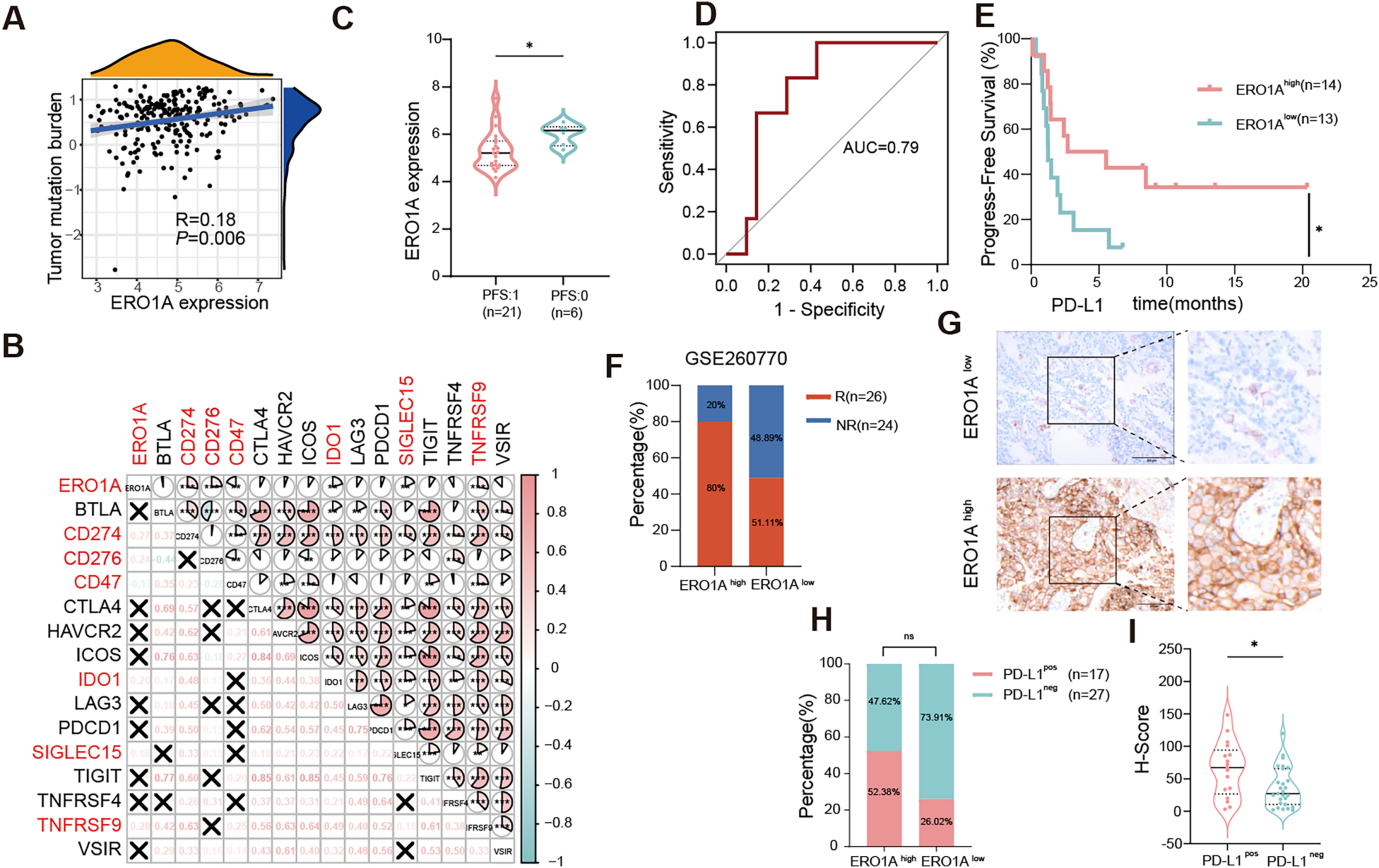
Prediction the role of ERO1A response to esLUAD immunotherapy. (A) Correlation analysis between ERO1A expression and TMB in the TCGA esLUAD cohort. *P* = 0.006. Spearman’s correlation test. (B) Heatmap plots showing the correlations of ERO1A mRNA and expressions of immune checkpoints in esLUAD, according to the TCGA-based analyses. Pearson’s correlation test. (C–E) GSE135222 external immunotherapy cohort validation confirms the high expression of ERO1A in patients responding to immunotherapy. (C)Violin plots showing ERO1A expression levels in patients with progressive disease (PFS = 1, *n* = 21) *vs* those with stable disease or better (PFS = 0, *n* = 6) from the GSE135222 cohort. *P* = 0.0153. Unpaired t test with Welch’s correction. (D) ROC curve analysis demonstrating the predictive value of ERO1A expression for immunotherapy response, with an area under the curve (AUC) of 0.79. (E) Kaplan-Meier survival curves comparing PFS between high and low ERO1A expression groups in immunotherapy patients. *P* = 0.0331. *P < 0.05. log-rank test. (F) Bar graph showing the percentage of ERO1A expression in R (*n* = 26) and NR (*n* = 24) groups from the GSE260770 cohort, including 50 early-stage NSCLC patients treated with sintilimab. (G) Representative immunohistochemical images of ERO1A and PD-L1 from our institutional esLUAD cohort. The right panel shows a magnified view of the boxed region. Scale bar: 200 μm. (H) Comparison of PD-L1 positivity rates between ERO1A-high and ERO1A-low esLUAD samples. ERO1A expression was evaluated by H-Score, and PD-L1 positivity was assessed by IHC using the SP263 antibody. “ns” indicates not significant (*P* > 0.05); the chi-square test yielded *P* = 0.074, suggesting a borderline significant trend toward higher PD-L1 positivity in ERO1A-high tumors. Chi-square test. (I) Violin plot comparing H-score of ERO1A between PD-L1 positive (*n* = 17) and negative (*n* = 27) groups in IHC staining. The analysis observes the PD-L1-positive group expresses higher levels of ERO1A than the PD-L1 negative group in IHC. *P* = 0.024. *P < 0.05. Mann Whitney test.

Given the association between ERO1A and an immune-activated TME, we further evaluated its potential as a predictive biomarker for immunotherapy response. We first assessed the correlation between ERO1A expression and established predictive markers, including tumor mutation burden (TMB) and immune checkpoint molecules. In the TCGA-esLUAD cohort, ERO1A expression positively correlated with TMB (Fig. 6A) and multiple inhibitory immune checkpoint molecules, including CD274 (which encodes PD-L1) (Fig. 6B), suggesting that high ERO1A expression may be associated with improved response to immunotherapy. To validate this hypothesis, we analyzed samples from the GEO immunotherapy cohort GSE135222, which includes advanced non-small cell lung cancer patients. ERO1A expression was significantly elevated in patients who responded to immunotherapy (non-recurrent group) (Fig. 6C). ROC analysis demonstrated that ERO1A expression strongly predicted immunotherapy recurrence (AUC = 0.79; Fig. 6D). Furthermore, survival analysis indicated that patients with high ERO1A expression had significantly prolonged progression-free survival (PFS) (hazard ratio, 2.59; 95% CI [1.04–6.43]; *P* = 0.033; Fig. 6E).

To determine whether ERO1A exhibits a similar predictive role in esLUAD, we analyzed an independent immunotherapy cohort (GSE260770) and found that high ERO1A expression was associated with improved treatment response (Fig. 6F). Furthermore, IHC validation in our institutional cohort revealed higher PD-L1 expression in ERO1A-high esLUAD samples (Fig. 6G). Statistical analysis showed a trend toward higher PD-L1 positivity in ERO1A-high patients, although this did not reach statistical significance (*P* = 0.074; Fig. 6H). Consistently, PD-L1 positive samples exhibited significantly higher ERO1A expression (*P* = 0.024; Fig. 6I). Collectively, these results suggest that ERO1A-high esLUAD patients are more likely to benefit from immunotherapy, supporting its potential role as a predictive biomarker for immune checkpoint inhibition in esLUAD.

### ERO1A expression correlates with enhanced sensitivity to targeted therapies in esLUAD

Based on prior pathway enrichment analyses indicating a strong association between high ERO1A expression and cellular proliferation, we hypothesized that ERO1A levels might influence the sensitivity of esLUAD patients to certain chemotherapeutic and targeted agents. We therefore predicted drug sensitivity based on the GDSC2 database for esLUAD patients stratified by ERO1A expression. A total of 97 drugs showed significantly differential sensitivity between the high and low ERO1A expression groups (Table S4). Among these, first- and second-generation EGFR-TKIs—gefitinib and afatinib—as well as the multi-target SRC/ABL/SFK inhibitor dasatinib exhibited significantly lower IC_50_ values in the high ERO1A group (Figs. S2A–S2C), suggesting enhanced sensitivity to these targeted agents in ERO1A-high patients.

Given the critical role of EGFR mutation as a therapeutic target in LUAD, we further examined the relationship between EGFR mutation status and ERO1A expression in our institutional cohort. IHC results revealed significantly higher ERO1A expression in EGFR-mutant esLUAD samples (Figs. S2D, S2E, *P* < 0.001). However, among EGFR-mutant patients treated with EGFR inhibitors, ERO1A expression did not differ significantly between those with and without disease progression (Fig. S2F). These findings suggest that high ERO1A expression may enhance sensitivity to certain targeted therapies and is associated with EGFR mutation status, though it may not predict DFS outcomes in EGFR-inhibitor-treated patients.

## Discussion

By integrating multi-omics data from public cohorts with immunohistochemical validation in an independent clinical series, we demonstrate that elevated ERO1A expression in esLUAD serves as a robust and independent predictor of early recurrence and mortality. Paradoxically, these aggressive ERO1A-high tumors concurrently exhibit an immune-activated TME associated with superior response to immune checkpoint blockade (ICB). These findings advocate for the refinement of current prognostic models and propose a practical clinical strategy: prioritizing peri-operative immunotherapy in ERO1A-high esLUAD, while considering conventional chemotherapy or targeted therapies for ERO1A-low cases.

Across multiple independent cohorts, high ERO1A expression was consistently associated with adverse clinicopathological features and reduced survival in esLUAD. This association remained significant after adjusting for age, sex, smoking history, and pathologic stage, underscoring the additive prognostic value of ERO1A beyond conventional parameters. These results align with previous pan-cancer studies and reports focused primarily on advanced NSCLC (Chen et al., 2024c), and are further supported by proteomic analyses from Zhou et al. (2021) and Hsu et al. (2016), which confirmed that high ERO1A expression predicts poor prognosis even in esLUAD. This collective evidence extends the prognostic significance of ERO1A to early-stage disease and supports its incorporation into risk-stratification frameworks.

Functionally, ERO1A has been implicated in diverse oncogenic processes across cancer types, including regulation of cell proliferation, apoptosis, angiogenesis, metastasis, and extracellular matrix remodeling (Shi et al., 2020; Voronkova et al., 2023; Chen et al., 2024c). In esLUAD, our bioinformatic analyses reveal that ERO1A is closely associated with cell cycle progression and DNA replication. This is further supported by a positive correlation with Ki67 expression, consistent with reports in NSCLC and breast cancer where ERO1A promotes proliferation through mechanisms such as dysregulation of Cyclin D1/CDK6 (Shi et al., 2020; Varone et al., 2025). Additionally, ERO1A may facilitate tumor progression through activation of pathways including Wnt/β-catenin (Xie et al., 2022), IL-6 signaling (Lei et al., 2020), calcium flux (Li et al., 2009; Chen et al., 2024b), and glycolytic metabolism (Sweef, Yang & Wang, 2022; Bassot et al., 2023). These cross-cancer comparisons highlight the conserved role of ERO1A as a multifunctional oncoprotein.

Contrary to reports in advanced NSCLC where ERO1A fosters an immunosuppressive microenvironment (Liu et al., 2021), ERO1A-high esLUAD tumors in our analysis displayed features suggestive of an immunologically active milieu, including enriched CD8^+^ T cell infiltration, reduced M2 macrophage polarization, increased mature tertiary lymphoid structures (mTLS), and positive correlations with PD-L1 expression and tumor mutational burden. Accordingly, in two independent immunotherapy-treated NSCLC cohorts, GSE135222 (advanced NSCLC) and GSE260770 (esLUAD), responders exhibited significantly higher ERO1A expression than non-responders, supporting the predictive potential of ERO1A specifically in lung adenocarcinoma.

A key question is why ERO1A-high tumors, despite an immune-activated phenotype, are associated with poor prognosis. First, consistent with previous studies, ERO1A promotes tumor proliferation and metastasis through multiple oncogenic pathways (Lei et al., 2020; Shi et al., 2020; Xie et al., 2022; Chen, Wang & Wang, 2022). Second, our immune infiltration analysis across multiple algorithms revealed that ERO1A expression correlates with distinct immune cell compositions. After excluding populations with inconsistent algorithm trends, we found that ERO1A positively correlates with M0 and M1 macrophages, Th1 cells, activated CD4^+^ memory T cells, and CD8^+^ memory T cells—all recognized for their roles in anti-tumor immunity and favorable immunotherapy response (Yin et al., 2021). Conversely, ERO1A negatively correlates with immunosuppressive populations including M2 macrophages, myeloid dendritic cells, and B cells (Hu et al., 2019). Thus, ERO1A-high tumors are characterized by increased infiltration of immune cells associated with anti-tumor immunity. However, our data also reveal that ERO1A expression positively correlates with multiple immune checkpoint molecules, including PD-L1, CD276, CD47, IDO1, and SIGLEC15 (Fig. 6B). IHC validation confirmed a trend toward higher PD-L1 positivity in ERO1A-high tumors (Figs. 6H, 6I). These findings suggest that infiltrating CD8^+^ T cells in ERO1A-high tumors may exist in a functionally suppressed state, limiting their anti-tumor activity despite their presence. Crucially, the observation that ERO1A-high patients show superior response in immunotherapy-treated cohorts, despite their poor prognosis in untreated settings, highlights the conceptual distinction between prognostic and predictive biomarkers. Our survival data establish ERO1A as a prognostic biomarker associated with aggressive tumor biology, while the immune microenvironment features suggest a predictive role for ICB benefit in LUAD.

Systematic assessment of the tumor microenvironment has emerged as a powerful approach to understand antitumor immunity and predict therapeutic outcomes. Recent studies have developed multi-omics integration tools to characterize immune heterogeneity and its impact on prognosis in lung adenocarcinoma (Sun et al., 2020). However, tools specifically tailored for early-stage LUAD remain limited. Here, we employed a multi-algorithm deconvolution strategy to comprehensively evaluate the relationship between ERO1A expression and immune cell infiltration in esLUAD. It is worth noting that in our immune infiltration analysis, we observed some inconsistencies across different deconvolution algorithms. For instance, while B cells showed consistent negative correlation with ERO1A across five algorithms (ABIS, ConsensusTME, EPIC, TIMER, and xCell), more heterogeneous populations such as cancer-associated fibroblasts (CAFs) exhibited opposing correlations when estimated by EPIC (positive) *vs* xCell (negative). Such discrepancies are commonly attributable to two factors. First, methodological differences: regression-based deconvolution methods (*e.g*., CIBERSORT, EPIC, quanTIseq) estimate absolute cell fractions based on reference expression matrices, whereas enrichment-based methods (*e.g*., xCell) calculate relative abundance scores through single-sample gene set enrichment analysis (ssGSEA) (Newman et al., 2015; Wolfram-Schauerte et al., 2025). Second, cell-type specificity: heterogeneous or plastic populations such as CAFs and macrophage subtypes lack universally accepted signature matrices and often share transcriptional programs with other lineages, making their estimation highly dependent on the reference dataset and algorithm used. To address these inherent biases, we focused on the overall immune context rather than isolated cell types, and performed experimental validation using immunohistochemistry on our independent cohort. This approach confirmed key associations at the protein level and provided spatial distribution patterns unattainable through bulk transcriptomic deconvolution, including B cell aggregates suggestive of tertiary lymphoid structures and CD8^+^ T-cell distribution patterns defining immune-inflamed, immune-excluded, or immune-desert phenotypes.

This divergence from the immunosuppressive role of ERO1A in metastatic settings may reflect disease stage-specific biology. We hypothesize that in esLUAD, ERO1A-mediated oxidative stress initially triggers an immune-activated microenvironment as a host response to oncogenic stress. However, as the disease progresses to advanced stages, chronic oxidative stress may promote immune cell exhaustion, recruitment of immunosuppressive populations, and upregulation of inhibitory molecules, ultimately establishing an immunosuppressive TME. This dynamic remodeling hypothesis is consistent with the known dual roles of other biomarkers; for instance, TGF-β exhibits tumor-suppressive effects in early stages but promotes invasion and immunosuppression in advanced disease (Amit & Deliang, 2010; Ikushima & Miyazono, 2010; Papageorgis & Stylianopoulos, 2015; Pickup, Owens & Moses, 2017). Differences in oncogenic drivers, cellular composition (*e.g*., tumor-intrinsic *vs* immune cell ERO1A expression), and immune profiling methodologies may also contribute to this discrepancy. Regardless of the underlying mechanisms, results from both external cohorts and our internal validation consistently position ERO1A as a promising predictive biomarker for ICB response in esLUAD.

Complementing these findings, *in silico* drug sensitivity analysis revealed that ERO1A-high tumors exhibit heightened resistance to the majority of conventional agents, while showing increased sensitivity to EGFR-TKIs (gefitinib and afatinib) and the multi-kinase inhibitor dasatinib. Notably, ERO1A has been identified as a poor prognostic indicator in EGFR-mutant non-small cell lung cancer (Voronkova et al., 2024). However, clinical validation within our EGFR-mutant cohort did not show significant association between ERO1A expression and survival outcomes following EGFR inhibitor therapy, suggesting rapid adaptive resistance *in vivo*. It is important to note that the GDSC2 database is derived from cancer cell lines and may not fully represent the drug response of esLUAD tumors in their native microenvironment; these findings should be considered exploratory, and validation in early-stage-specific models or patient-derived samples is needed in future studies. Future studies employing systematic experimental approaches (Wang et al., 2020) are warranted to confirm the predictive value of ERO1A for targeted therapy response.

Several limitations merit consideration. First, the retrospective nature of this study, the predominance of Western populations in public databases, and the relatively modest size of our IHC validation cohort (*n* = 99) collectively necessitate validation in larger, multi-ethnic prospective cohorts. Second, although bioinformatic inferences are robust, mechanistic validation using CRISPR-based or inducible models is essential to establish causality. Third, while our analysis utilized two lung cancer-specific immunotherapy cohorts (GSE135222 and GSE260770), these datasets are relatively small and include predominantly advanced-stage patients. Therefore, the predictive value of ERO1A for immunotherapy response, particularly in early-stage LUAD, should be prospectively assessed in the context of randomized neoadjuvant trials designed specifically for this patient population.

## Conclusions

In conclusion, ERO1A expression stratifies esLUAD into two distinct subsets: an immune-activated, chemotherapy-resistant subgroup amenable to immunotherapy, and an immune-quiescent subgroup potentially responsive to conventional agents. Its role as a prognostic biomarker in esLUAD is particularly noteworthy. Future investigations should explore ERO1A as a potential combinatorial target to enhance immunotherapy response and as a biomarker of therapeutic resistance, which could further refine treatment stratification. Incorporating ERO1A evaluation into standard pathologic assessment could guide peri-operative therapeutic strategies, advancing the paradigm of precision medicine in early-stage lung cancer.

## Supplemental Information

10.7717/peerj.21347/supp-1 Supplemental Information 1 TCGA early-stage LUAD cohort. De-identified clinical data for the TCGA early-stage LUAD cohort (*n* = 239) used for bioinformatic analyses in this study. This is a processed subset of the publicly available data from The Cancer Genome Atlas (TCGA).

10.7717/peerj.21347/supp-2 Supplemental Information 2 Clinical specimens. Raw and de-identified clinical data for the institutional cohort of 99 patients with early-stage lung adenocarcinoma (esLUAD) used in this study.

10.7717/peerj.21347/supp-3 Supplemental Information 3 Tissue specimens from 153 patients who underwent pulmonary resection surgery at Guangzhou First People’s Hospital between 2022 and 2023.

10.7717/peerj.21347/supp-4 Supplemental Information 4 Supplementary Information. A. Supplementary Note Notes S1. Supplementary Method. Immunohistochemical (IHC) Staining and Scoring. B． Supplementary Table Tables S1 to S4 Table S1. Baseline demographic and disease characteristics of the included patients with esLUAD. Table S2. Immune contexture characterized by CD8^+^ T cells, CD68^+^ macrophages and FOXP3⁺ Tregs in esLUAD patients stratified by ERO1A expression. Table S3. Protein expressions of ERO1A in HPA database. Table S4. Analysis of drug sensitivity in relation to ERO1A expression levels in esLUAD patients. C. Supplementary Figure Figures S1 to S2.

10.7717/peerj.21347/supp-5 Supplemental Information 5 Code. 1. immune checkpoint.R: This code produces a correlation plot (corpot.pdf) showing the correlation between ERO1A and immune checkpoint genes. 2. enrichment.R: This code produces a bar plot for GO enrichment. The legend should describe the bar plot. 3. GSEA.R: This code produces multiple GSEA plots (*e.g*., enrichment plots, dot plots, ridge plots). 4. Gene Screening.R: This code produces Kaplan-Meier survival curves, ROC curves, and machine learning results (LASSO, RF, SVM). 5. correlation.R: This code produces scatter plots for ERO1A and other genes. 6. Immune Infiltration.R: This code produces a correlation plot (immune cells *vs* ERO1A), scatter plots for immune cells.

## Additional Information and Declarations

### Data Availability

The following information was supplied regarding data availability:

The data is available at The Cancer Genome Atlas (TCGA), project: TCGA-LUAD, and accessed and processed *via* the Sangerbox website (http://www.sangerbox.com).

Additional validation datasets are available at NCBI GEO: GSE72094, GSE135222 and GSE260770.

The code is available in the Supplemental Files.
