# Supplementary material for: ERO1A as a novel biomarker for risk stratification and immunotherapeutic guidance in early-stage lung adenocarcinoma
Source: PeerJ. 2026 Jun 17;14:e21347. doi: 10.7717/peerj.21347 (PMC13282948; doi:10.7717/peerj.21347)
Supplement: Supplemental Information 4 — A. Supplementary Note Notes S1. Supplementary Method. Immunohistochemical (IHC) Staining and Scoring. B． Supplementary Table Tables S1 to S4 Table S1. Baseline demographic and disease characteristics of the included patients with esLUAD. Table S2. Immune contexture characterized by CD8+ T cells, CD68+ macrophages and FOXP3⁺ Tregs in esLUAD patients stratified by ERO1A expression. Table S3. Protein expressions of ERO1A in HPA database. Table S4. Analysis of drug sensitivity in relation to ERO1A expression levels in esLUAD patients. C. Supplementary Figure Figures S1 to S2. [file peerj-14-21347-s004.docx]

**Article Title: ERO1A as a Novel Biomarker for Risk Stratification and Immunotherapeutic Guidance in Early-Stage Lung Adenocarcinoma**

**Authors:** Wangyu Chen^1*^, Haiming Li^2*^, Jie Zeng^1^, Guijing Xie^1^, Zhibo Chen^1^, Xie Xu^1^, Wenjie Bai^1^, Gang Xiao^1,3†^, Jianjiang Xie^1,2†^

**Affiliation:**

^1^Department of Thoracic Surgery, Guangzhou First People's Hospital, South China University of Technology, Guangzhou 510180, China.

^2^Department of Thoracic Surgery, Guangzhou First People's Hospital, Guangdong Medical University, Guangzhou 510180, China.

^3^Center for Medical Research on Innovation and Translation, Guangzhou First People's Hospital, South China University of Technology, Guangzhou 510180, China.

**^†^ Corresponding author:** [eyxiaogang@scut.edu.cn](mailto:eyxiaogang@scut.edu.cn) (Gang Xiao); [eyxiejianjiang@scut.edu.cn](mailto:eyxiaogang@scut.edu.cn), +86 20-81048236 (Jianjiang Xie, lead contact).

**Supplementary Information**

**This files includes:**

1. **Supplementary Note**

Notes S1

Note S1. Supplementary Method. Immunohistochemical (IHC) Staining and Scoring.

1. **Supplementary Table**

Tables S1 to S4

Table S1. Baseline demographic and disease characteristics of the included patients with esLUAD.

Table S2. Immune contexture characterized by CD8⁺ T cells, CD68⁺ macrophages and FOXP3⁺ Tregs in esLUAD patients stratified by ERO1A expression.

Table S3. Protein expressions of ERO1A in HPA database.

Table S4. Analysis of drug sensitivity in relation to ERO1A expression levels in esLUAD patients.

1. **Supplementary Figure**

Figures S1 to S2

1. **Supplementary Note**

Notes S1. Supplementary Method: Immunohistochemical (IHC) Staining and Scoring

IHC was conducted on formalin-fixed, paraffin-embedded (FFPE) tissue sections. The tissue slides were first baked (60°C, 1h), then deparaffinized and hydrated, followed by antigen retrieval in buffer. Endogenous peroxidase activity was blocked with 3% H_2_O_2_ solution. Sections were incubated with primary antibodies overnight at 4°C, then incubated with secondary antibodies at room temperature for 30 minutes. Visualization was performed using 3,3'-diaminobenzidine (DAB) tetrahydrochloride, and counterstaining was done with hematoxylin. Finally, the sections were dehydrated, cleared, and sealed with neutral resin. The slides were observed under a microscope, with three fields of view photographed for each group. Image analysis was performed using Image J software.

Staining intensity was scored and graded as 0 (negative), 1 (weak), 2 (moderate), and 3 (strong). The ERO1A level of each sample was calculated based on staining distribution and intensity using the formula: H-score = (percentage of weak intensity × 1) + (percentage of moderate intensity × 2) + (percentage of strong intensity × 3). ERO1A expression was divided into high and low groups according to the median H-score. B cell grading was categorized as None (minimal CD20), Diffuse (scattered cells), immature TLS (imTLS, dense CD20+ aggregate without pale core), mature TLS (mTLS, organized structure with central CD20- zone). The classification of CD8 cells was based on their spatial distribution, defining three phenotypes: Desert, Excluded, and Inflamed. These scores were independently determined by two experienced pathologists who were blinded to clinicopathological data. The summarized data for these immune cell subsets, stratified by ERO1A expression, are presented in Table S.2.

The antibodies used in this study were as follows: primary antibodies—ERO1A (1:100, Abcam, ab177156); CD8 (cytotoxic T cells, ZS, ZA-0508); CD68 (macrophages, ZS, ZM-0060-6.0); CD20 (B cells, ZS, TA800394); FOXP3 (regulatory T cells, ZS, ZA-0682-3.0). The secondary antibody was the Anti-Rabbit and Mouse HRP-DAB IHC detection kit (2-step).

Additionally, we obtained PD-L1 antibody (Ventana SP263) images and measured PD-L1 expression levels through immunohistochemistry from the pathology department. For the evaluation of PD-L1, tumor proportion score >1% was considered positive. Images and expression values of KI-67 were also retrieved from the pathology department.

1. **Supplementary Table**

**Supplementary Table 1.**

| Table S1. Baseline demographic and disease characteristics of the included patients with esLUAD. | | | | |
| --- | --- | --- | --- | --- |
| **Clinical characteristic** | ERO1A^low^(N=49) | ERO1A^high^(N=50) | Total(N=99) | P |
| **ERO1A** |  |  |  |  |
| Mean±SD | 18.18±12.41 | 74.89±25.83 | 46.82±34.94 |  |
| Median[min-max] | 18.51[1.42,36.86] | 71.25[37.04,148.52] | 37.04[1.42,148.52] |  |
| **sex** |  |  |  | 0.17 |
| Female | 34(34.34%) | 27(27.27%) | 61(61.62%) |  |
| Male | 15(15.15%) | 23(23.23%) | 38(38.38%) |  |
| **age(years)** |  |  |  |  |
| Mean±SD | 60.98±7.84 | 62.60±11.15 | 61.80±9.64 |  |
| Median[min-max] | 60.00[43.00,76.00] | 64.00[43.00,88.00] | 62.00[43.00,88.00] |  |
| **height(cm)** |  |  |  |  |
| Mean±SD | 159.11±8.44 | 160.31±8.21 | 159.69±8.29 |  |
| Median[min-max] | 159.50[145.00,176.00] | 161.00[145.00,173.00] | 160.00[145.00,176.00] |  |
| **weight(kg)** |  |  |  |  |
| Mean±SD | 56.27±9.82 | 60.27±11.65 | 58.29±10.92 |  |
| Median[min-max] | 56.00[40.00,80.00] | 60.00[40.00,82.00] | 59.00[40.00,82.00] |  |
| **stage** |  |  |  | 0.33 |
| TisN0M0 | 0(0.0e+0%) | 1(1.01%) | 1(1.01%) |  |
| IA | 37(37.37%) | 29(29.29%) | 66(66.67%) |  |
| IB | 11(11.11%) | 18(18.18%) | 29(29.29%) |  |
| ⅡA | 0(0.0e+0%) | 1(1.01%) | 1(1.01%) |  |
| IIB | 1(1.01%) | 1(1.01%) | 2(2.02%) |  |
| **stage_T** |  |  |  | 0.28 |
| T1mi | 1(1.01%) | 1(1.01%) | 2(2.02%) |  |
| Tis | 0(0.0e+0%) | 1(1.01%) | 1(1.01%) |  |
| T1 | 37(37.37%) | 28(28.28%) | 65(65.66%) |  |
| T2 | 10(10.10%) | 19(19.19%) | 29(29.29%) |  |
| T3 | 1(1.01%) | 1(1.01%) | 2(2.02%) |  |
| **smoking** |  |  |  | 0.23 |
| Ever | 9(9.09%) | 10(10.10%) | 19(19.19%) |  |
| Stop | 1(1.01%) | 5(5.05%) | 6(6.06%) |  |
| Never | 39(39.39%) | 35(35.35%) | 74(74.75%) |  |
| **CEA** |  |  |  |  |
| Mean±SD | 2.40±1.71 | 4.27±7.77 | 3.35±5.73 |  |
| Median[min-max] | 2.06[0.45,7.49] | 2.69[0.83,53.60] | 2.30[0.45,53.60] |  |
| **CYFRA21-1** |  |  |  |  |
| Mean±SD | 2.78±1.80 | 2.68±1.15 | 2.73±1.50 |  |
| Median[min-max] | 2.33[0.89,11.49] | 2.51[1.02,7.06] | 2.42[0.89,11.49] |  |
| **NSE** |  |  |  |  |
| Mean±SD | 11.94±4.45 | 12.72±4.24 | 12.34±4.34 |  |
| Median[min-max] | 11.10[0.44,32.40] | 11.70[1.49,25.70] | 11.30[0.44,32.40] |  |
| **SCCA** |  |  |  |  |
| Mean±SD | 1.31±0.60 | 1.40±0.67 | 1.36±0.64 |  |
| Median[min-max] | 1.16[0.38,3.08] | 1.18[0.47,3.55] | 1.17[0.38,3.55] |  |
| **adjuvant therapy** |  |  |  | 1.30E-04 |
| No | 43(43.43%) | 25(25.25%) | 68(68.69%) |  |
| Yes | 6(6.06%) | 25(25.25%) | 31(31.31%) |  |
| **EGFR** |  |  |  | 1.60E-03 |
| Mut | 2(2.02%) | 15(15.15%) | 17(17.17%) |  |
| WT | 47(47.47%) | 35(35.35%) | 82(82.83%) |  |
| **postoperative immunotherapy** |  |  |  | 0.07 |
| No | 49(49.49%) | 45(45.45%) | 94(94.95%) |  |
| Yes | 0(0.0e+0%) | 5(5.05%) | 5(5.05%) |  |
| **Ki-67(%)** |  |  |  |  |
| Mean±SD | 7.67±4.71 | 22.26±23.94 | 15.04±18.74 |  |
| Median[min-max] | 5.00[2.00,20.00] | 10.00[1.00,95.00] | 10.00[1.00,95.00] |  |
| **KI-67_cluster** |  |  |  | 4.60E-05 |
| L (<10%) | 41(44.09%) | 25(26.88%) | 66(70.97%) |  |
| M (10%-20%) | 5(5.38%) | 6(6.45%) | 11(11.83%) |  |
| H (>20%) | 0(0.0e+0%) | 16(17.20%) | 16(17.20%) |  |
| **DFS_status** |  |  |  | 7.80E-03 |
| No | 46(51.11%) | 36(40.00%) | 82(91.11%) |  |
| Yes | 0(0.0e+0%) | 8(8.89%) | 8(8.89%) |  |
| **DFS_time(days)** |  |  |  |  |
| Mean±SD | 994.04±144.93 | 883.40±221.86 | 939.92±193.67 |  |
| Median[min-max] | 917.00[742.00,1208.00] | 895.00[18.00,1201.00] | 906.50[18.00,1208.00] |  |
| **OS_time(days)** |  |  |  |  |
| Mean±SD | 994.04±144.93 | 950.73±159.41 | 972.86±152.90 |  |
| Median[min-max] | 917.00[742.00,1208.00] | 900.00[557.00,1201.00] | 912.50[557.00,1208.00] |  |
| **PD-L1** |  |  |  | 0.14 |
| Negative | 17(38.64%) | 10(22.73%) | 27(61.36%) |  |
| Positive | 6(13.64%) | 11(25.00%) | 17(38.64%) |  |
| **TLS** |  |  |  | 0.03 |
| None | 13(13.40%) | 12(12.37%) | 25(25.77%) |  |
| Diffuse | 14(14.43%) | 4(4.12%) | 18(18.56%) |  |
| imTLS | 15(15.46%) | 17(17.53%) | 32(32.99%) |  |
| mTLS | 7(7.22%) | 15(15.46%) | 22(22.68%) |  |
| **CD8** |  |  |  | 0.04 |
| Desert | 5(5.15%) | 6(6.19%) | 11(11.34%) |  |
| Excluded | 19(19.59%) | 8(8.25%) | 27(27.84%) |  |
| Inflamed | 24(24.74%) | 35(36.08%) | 59(60.82%) |  |

ERO1A, Endoplasmic Reticulum Oxidoreductase 1 Alpha; esLUAD, early-stage lung adenocarcinoma; DFS, Disease-Free Survival; OS, Overall Survival; PD-L1, Programmed Death-Ligand 1; TLS, Tertiary Lymphoid Structure; imTLS, immature Tertiary Lymphoid Structure; mTLS, mature Tertiary Lymphoid Structure.

**Supplementary Table 2.**

| Table S2. Immune contexture characterized by CD8⁺ T cells, CD68⁺ macrophages and FOXP3⁺ Tregs in esLUAD patients stratified by ERO1A expression. | | | |
| --- | --- | --- | --- |
| Characteristics | ERO1A^low^(N=30) | ERO1A^high^(N=27) | Total(N=57) |
| **CD8** |  |  |  |
| Mean±SD | 289.47±249.74 | 430.30±315.38 | 356.18±288.98 |
| Median[min-max] | 210.50[21.00,886.00] | 326.00[31.00,1432.00] | 273.00[21.00,1432.00] |
| **CD68** |  |  |  |
| Mean±SD | 301.87±217.51 | 282.52±291.63 | 292.70±253.14 |
| Median[min-max] | 252.50[16.00,869.00] | 235.00[13.00,1184.00] | 235.00[13.00,1184.00] |
| **FOXP3** |  |  |  |
| Mean±SD | 49.60±42.30 | 58.63±49.80 | 53.88±45.81 |
| Median[min-max] | 33.00[2.00,131.00] | 40.00[1.00,171.00] | 35.00[1.00,171.00] |

**Supplementary Table 3.**

| Table S3. Protein expressions of ERO1A in HPA database. | | | | | | | | |
| --- | --- | --- | --- | --- | --- | --- | --- | --- |
| Patient ID | Gender | Age | Histology | staining | Intensity | Quantity | Location | Antibody |
| 2003 | Female | 61 | Adenocarcinoma, NOS | high | Strong | 75%-25% | Cytoplasmic/ membranous | HPA026653 |
| 4923 | Male | 57 | Adenocarcinoma, NOS | high | Strong | 75%-25% | Cytoplasmic/ membranous | HPA026653 |
| 1907 | Male | 73 | Adenocarcinoma, NOS | Medium | Moderate | 75%-25% | Cytoplasmic/ membranous | HPA026653 |
| 4208 | Male | 75 | Adenocarcinoma, NOS | low | weak | 75%-25% | Cytoplasmic/ membranous | HPA026653 |
| 1932 | Female | 57 | Adenocarcinoma, NOS | low | Moderate | <25% | Cytoplasmic/ membranous | HPA026653 |
| 3144 | Female | 73 | Adenocarcinoma, NOS | Medium | Moderate | >75% | Cytoplasmic/ membranous | HPA026653 |
| 2003 | Female | 61 | Adenocarcinoma, NOS | Medium | Strong | <25% | Cytoplasmic/ membranous | HPA030053 |
| 4923 | Male | 57 | Adenocarcinoma, NOS | not-detect | weak | <25% | Cytoplasmic/ membranous | HPA030053 |
| 1907 | Male | 73 | Adenocarcinoma, NOS | Medium | Strong | <25% | Cytoplasmic/ membranous | HPA030053 |
| 4208 | Male | 75 | Adenocarcinoma, NOS | not-detect | weak | <25% | Cytoplasmic/ membranous | HPA030053 |
| 1932 | Female | 57 | Adenocarcinoma, NOS | Medium | Strong | <25% | Cytoplasmic/ membranous | HPA030053 |
| 3144 | Female | 73 | Adenocarcinoma, NOS | Medium | Moderate | 75%-25% | Cytoplasmic/ membranous | HPA030053 |
| 3391 | Female | 70 | Adenocarcinoma, NOS | Medium | Moderate | >75% | Cytoplasmic/ membranous | CAB034294 |
| 3003 | Mela | 49 | Adenocarcinoma, NOS | low | weak | >75% | Cytoplasmic/ membranous | CAB034294 |
| 2041 | Female | 51 | Adenocarcinoma, NOS | Medium | Moderate | >75% | Cytoplasmic/ membranous | CAB034294 |
| 2403 | Female | 65 | Adenocarcinoma, NOS | Medium | Moderate | >75% | Cytoplasmic/ membranous | CAB034294 |
| 1847 | Mela | 64 | Adenocarcinoma, NOS | Medium | Moderate | >75% | Cytoplasmic/ membranous | CAB034294 |
| 2222 | Mela | 59 | Adenocarcinoma, NOS | Medium | Moderate | >75% | Cytoplasmic/ membranous | CAB034294 |

ERO1A, Endoplasmic Reticulum Oxidoreductase 1 Alpha; HPA, Human Protein Atlas; NOS, Not Otherwise Specified.

**Supplementary Table 4.**

| Table S4. Analysis of drug sensitivity in relation to ERO1A expression levels in esLUAD patients. | | |
| --- | --- | --- |
| Drug | Type | More Sensitive in high/low Group |
| WZ4003 | Targeted Therapy | low |
| Wnt.C59 | Targeted Therapy | low |
| Vorinostat | Targeted Therapy | low |
| Venetoclax | Targeted Therapy | low |
| Uprosertib | Targeted Therapy | low |
| Tamoxifen | Targeted Therapy | low |
| Talazoparib | Targeted Therapy | low |
| TAF1_5496 | Targeted Therapy | low |
| Sorafenib | Targeted Therapy | low |
| Sinularin | Targeted Therapy | low |
| SB505124 | Targeted Therapy | low |
| Sabutoclax | Targeted Therapy | low |
| RO.3306 | Targeted Therapy | low |
| Rapamycin | Targeted Therapy | low |
| Picolinici.acid | Targeted Therapy | low |
| PFI3 | Targeted Therapy | low |
| PF.4708671 | Targeted Therapy | low |
| Pevonedistat | Targeted Therapy | low |
| PD173074 | Targeted Therapy | low |
| PCI.34051 | Targeted Therapy | low |
| Palbociclib | Targeted Therapy | low |
| PAK_5339 | Targeted Therapy | low |
| P22077 | Targeted Therapy | low |
| OTX015 | Targeted Therapy | low |
| OSI.027 | Targeted Therapy | low |
| Olaparib | Targeted Therapy | low |
| OF.1 | Targeted Therapy | low |
| NVP.ADW742 | Targeted Therapy | low |
| Nutlin.3a | Targeted Therapy | low |
| Niraparib | Targeted Therapy | low |
| Nilotinib | Targeted Therapy | low |
| ML323 | Targeted Therapy | low |
| MK.2206 | Targeted Therapy | low |
| MIRA.1 | Targeted Therapy | low |
| LY2109761 | Targeted Therapy | low |
| LJI308 | Targeted Therapy | low |
| Linsitinib | Targeted Therapy | low |
| LGK974 | Targeted Therapy | low |
| KRAS..G12C..Inhibitor.12 | Targeted Therapy | low |
| JQ1 | Targeted Therapy | low |
| IRAK4_4710 | Targeted Therapy | low |
| Ibrutinib | Targeted Therapy | low |
| I.BRD9 | Targeted Therapy | low |
| GSK591 | Targeted Therapy | low |
| GSK343 | Targeted Therapy | low |
| GSK269962A | Targeted Therapy | low |
| GSK2606414 | Targeted Therapy | low |
| GSK2578215A | Targeted Therapy | low |
| GSK1904529A | Targeted Therapy | low |
| GNE.317 | Targeted Therapy | low |
| Gefitinib | Targeted Therapy | high |
| GDC0810 | Targeted Therapy | low |
| Gallibiscoquinazole | Targeted Therapy | low |
| Fulvestrant | Targeted Therapy | low |
| EPZ5676 | Targeted Therapy | low |
| EPZ004777 | Targeted Therapy | low |
| Elephantin | Targeted Therapy | low |
| Eg5_9814 | Targeted Therapy | low |
| Doramapimod | Targeted Therapy | low |
| Dinaciclib | Targeted Therapy | low |
| Dihydrorotenone | Targeted Therapy | low |
| Dasatinib | Targeted Therapy | high |
| Daporinad | Targeted Therapy | low |
| Dactolisib | Targeted Therapy | low |
| Dabrafenib | Targeted Therapy | low |
| CZC24832 | Targeted Therapy | low |
| CDK9_5576 | Targeted Therapy | low |
| CDK9_5038 | Targeted Therapy | low |
| Buparlisib | Targeted Therapy | low |
| BMS.754807 | Targeted Therapy | low |
| BMS.345541 | Targeted Therapy | low |
| BIBR.1532 | Targeted Therapy | low |
| AZD8055 | Targeted Therapy | low |
| AZD5991 | Targeted Therapy | low |
| AZD5363 | Targeted Therapy | low |
| AZD5153 | Targeted Therapy | low |
| AZD4547 | Targeted Therapy | low |
| AZD2014 | Targeted Therapy | low |
| AZD1332 | Targeted Therapy | low |
| AZD1208 | Targeted Therapy | low |
| AZ6102 | Targeted Therapy | low |
| Axitinib | Targeted Therapy | low |
| AT13148 | Targeted Therapy | low |
| AGI.6780 | Targeted Therapy | low |
| AGI.5198 | Targeted Therapy | low |
| Afuresertib | Targeted Therapy | low |
| Afatinib | Targeted Therapy | high |
| Zoledronate | Chemotherapy | low |
| Temozolomide | Chemotherapy | low |
| Oxaliplatin | Chemotherapy | low |
| Epirubicin | Chemotherapy | low |
| Dactinomycin | Chemotherapy | low |
| Cyclophosphamide | Chemotherapy | low |
| Carmustine | Chemotherapy | low |
| Leflunomide | Immunotherapy | low |
| JAK1_8709 | Immunotherapy | low |
| JAK_8517 | Immunotherapy | low |

ERO1A, Endoplasmic Reticulum Oxidoreductase 1 Alpha; esLUAD, early-stage Lung Adenocarcinoma.

1. **Supplementary Figure**

**
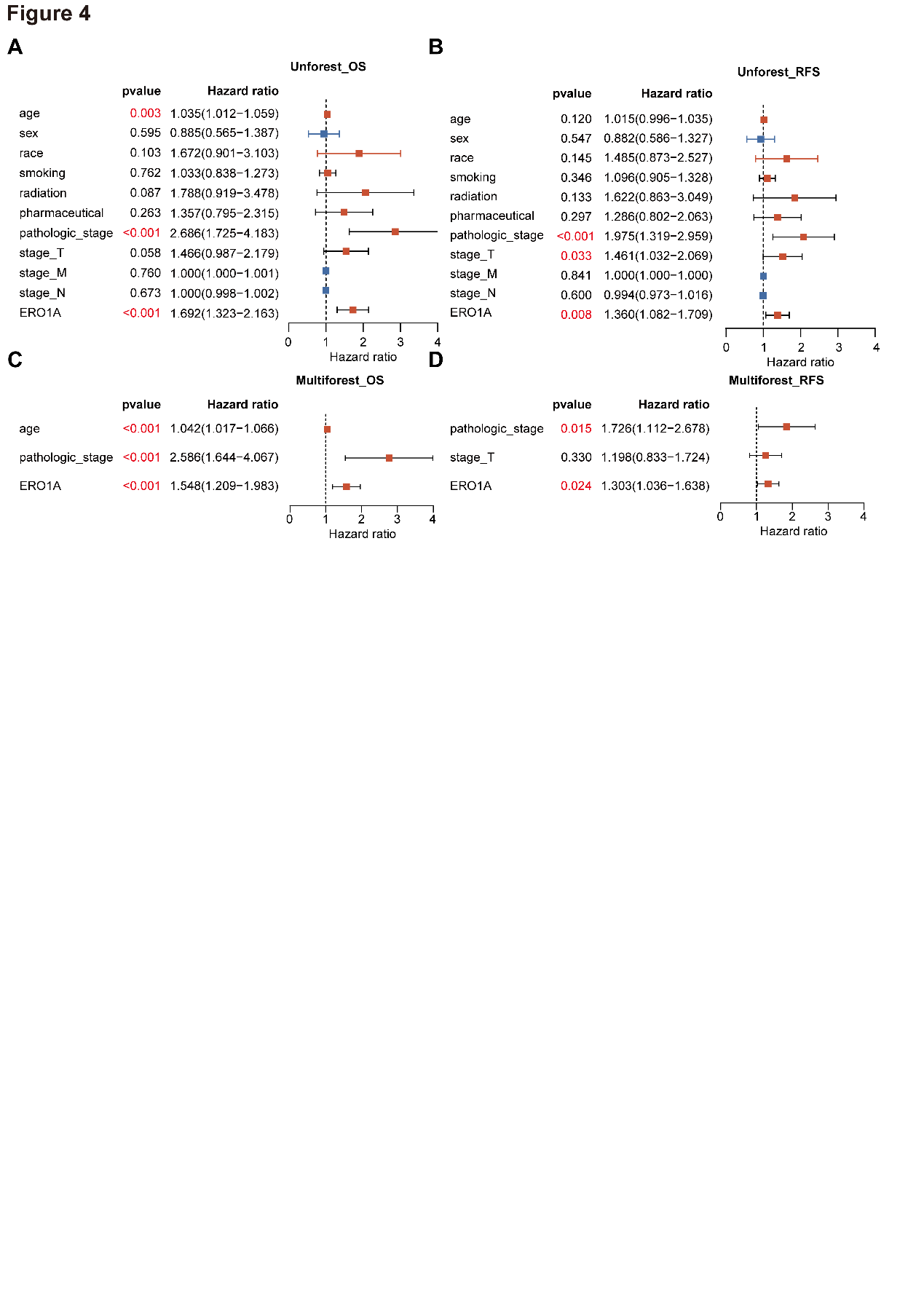
**

**Fig. S1. ERO1A is an independent prognostic factor in esLUAD.**

Forest plots of Cox regression analyses in the TCGA-esLUAD cohort.

(A) Univariate analysis for overall survival (OS).

(B) Univariate analysis for relapse-free survival (RFS).

(C) Multivariate analysis for OS.

(D) Multivariate analysis for RFS.

Note: Multivariate models included all variables that were significant (P < 0.05) in the univariate analysis.

Abbreviations: esLUAD, early-stage lung adenocarcinoma; OS, overall survival; DFS, disease-free survival.


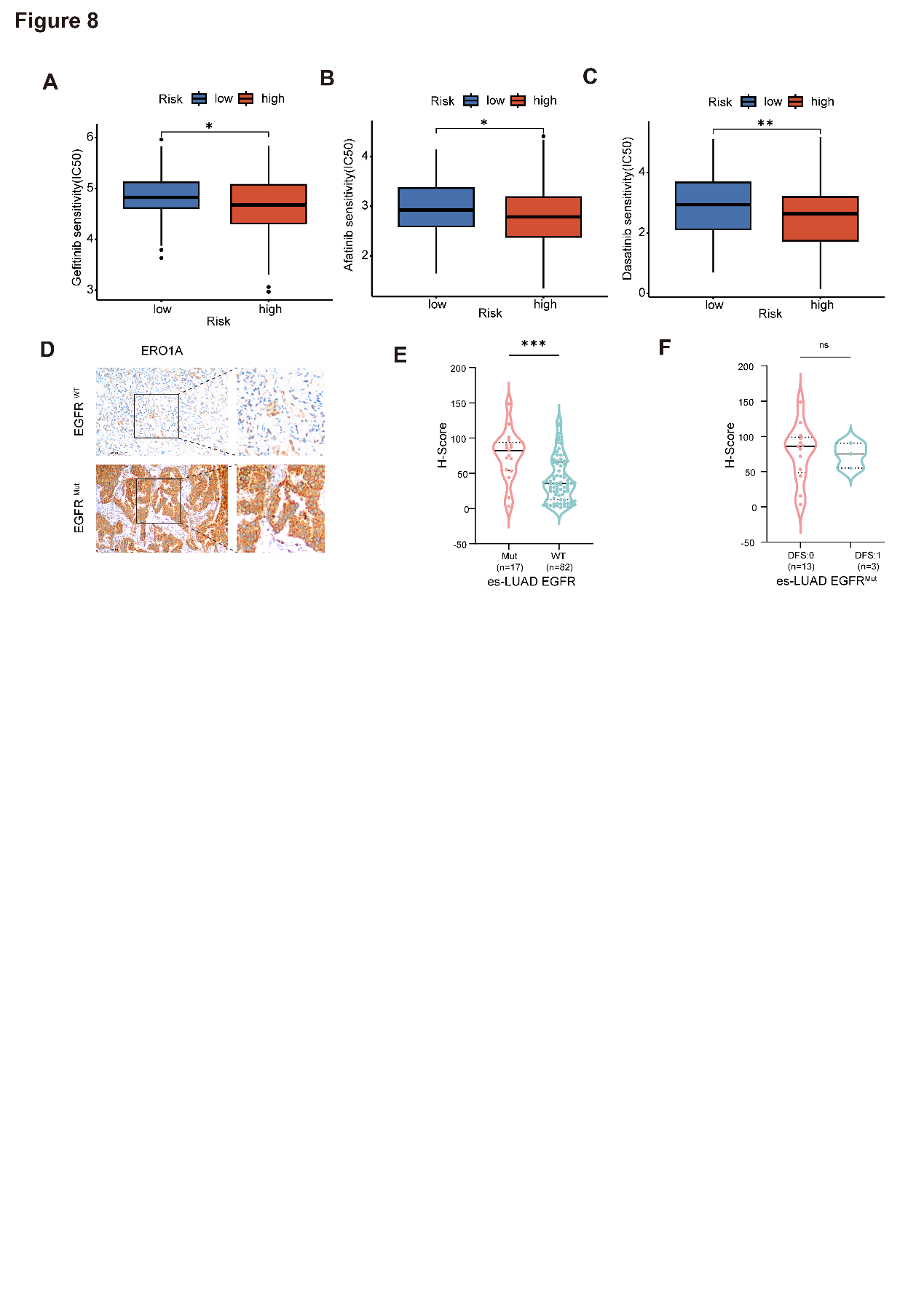
 **Fig. S2. Analysis of drug sensitivity in relation to ERO1A expression levels in esLUAD patients.**

(A-C) Box plots illustrating the IC50 values of different drugs in groups with high and low expression of ERO1A. The red indicates high expression of ERO1A and the blue for the low expression. (A) Gefitinib. P=0.03, (B) Afatinib. P=0.04., (C) Dasatinib. P= 0.008. Wilcoxon rank-sum test.

(D) Immunohistochemical staining of ERO1A protein in esLUAD tissue samples, comparing EGFR Mut and WT cases. The images show varying levels of ERO1A expression. Scale bar: 200 μm.

(E) Violin plot depicting the distribution of H-Score for ERO1A expression, comparing EGFR Mut (n=17) and EGFR WT (n=82) patient groups. The data reveals a significant upregulation of ERO1A in EGFR Mut patients. ***P <0.001. Mann Whitney test.

(F) Violin plot comparing H-Scores between esLUAD patients with EGFR Mut who experienced disease recurrence (n=3) and those without recurrence (n=13). P=0.8924. ns, not signiﬁcant. unpaired t test.

Abbreviations: IC50, half-maximal inhibitory concentration; EGFR, epidermal growth factor receptor; Mut, mutations; WT, Wild-Type.
